# Exosomes secreted by endothelial cells derived from human induced pluripotent stem cells improve recovery from myocardial infarction in mice

**DOI:** 10.1186/s13287-023-03462-w

**Published:** 2023-09-29

**Authors:** Hao Li, Lu Wang, Teng Ma, Zhongmin Liu, Ling Gao

**Affiliations:** 1 Translational Medical Center for Stem Cell Therapy and Institutes for Regenerative Medicine, Shanghai East Hospital, Tongji University School of Medicine, 1800 Yuntai Rd., Shanghai, 200123 China; 2 Department of Cardiovascular and Thoracic Surgery, Shanghai East Hospital, Tongji University School of Medicine, Shanghai, 200120 China; 3 Shanghai Institute of Stem Cell Research and Clinical Translation, Shanghai East Hospital, Tongji University, Shanghai, 200120 China; 4 Shanghai Engineering Research Center for Stem Cell Clinical Treatment, Shanghai, 200123 China

**Keywords:** hiPSC-ECs, Exosomes, Ca^2+^ homeostasis, miR-100-5p

## Abstract

**Background:**

Human induced pluripotent stem cell-derived endothelial cells (hiPSC-ECs) exhibit the potential to repair the injured heart after myocardial infarction (MI) by promoting neovascularization and cardiomyocyte survival. However, because of the low cellular retention and poor engraftment efficacy, cell therapy of MI is partly mediated by exosomes secreted from the transplanted cells. In this study, we investigated whether exosomes secreted from hiPSC-ECs could become a promising acellular approach to repair the infarcted heart after MI and elucidated the underlying protective mechanism.

**Methods:**

The hiPSC-ECs were differentiated, and exosomes were isolated in vitro. Then, hiPSC-EC exosomes were delivered by intramyocardial injection in a murine MI model in vivo. Echocardiography, combined with hemodynamic measurement, histological examination, Ca^2+^ transient and cell shortening assessment, and Western blot, was used to determine the protective effects of hiPSC-EC exosomes on the infarcted heart. Furthermore, microRNA sequencing, luciferase activity assay, and microRNA gain–loss function experiments were performed to investigate the enriched microRNA and its role in exosome-mediated effects.

**Results:**

In vitro, the hiPSC-EC exosomes enhanced intracellular Ca^2+^ transients, increased ATP content, and improved cell survival to protect cardiomyocytes from oxygen–glucose deprivation injury. Congruously, hiPSC-EC exosome administration in vivo improved the myocardial contractile function and attenuated the harmful left ventricular remodeling after MI without increasing the frequency of arrhythmias. Mechanistically, the hiPSC-EC exosomes notably rescued the protein expression and function of the sarcoplasmic reticulum Ca^2+^ ATPase 2a (SERCA-2a) and ryanodine receptor 2 (RyR-2) to maintain intracellular Ca^2+^ homeostasis and increase cardiomyocyte contraction after MI. The microRNA sequencing showed that miR-100-5p was the most abundant microRNA in exosomes. miR-100-5p could target protein phosphatase 1β (PP-1β) to enhance phospholamban (PLB) phosphorylation at Ser^16^ and subsequent SERCA activity, which contributes to the hiPSC-EC exosome-exerted cytoprotective effects on maintaining intracellular Ca^2+^ homeostasis and promoting cardiomyocyte survival.

**Conclusion:**

The hiPSC-EC exosomes maintain cardiomyocyte Ca^2+^ homeostasis to improve myocardial recovery after MI, which may provide an acellular therapeutic option for myocardial injury.

**Supplementary Information:**

The online version contains supplementary material available at 10.1186/s13287-023-03462-w.

## Introduction

Myocardial infarction (MI) causes irreversible cell death and may lead to heart failure (HF). It is the leading cause of morbidity and mortality worldwide [1–4]. Cell therapy, such as human induced pluripotent stem cell-derived endothelial cells (hiPSC-ECs), has emerged as a potential approach to repairing the infarcted heart [5, 6]. There is convincing evidence that iPSC-ECs (human and swine origin) could act as a regenerative cargo to protect the heart against ischemic injury by promoting neovascularization and cardiomyocyte survival [5–7]. However, low cellular retention and poor engraftment efficacy limit the therapeutic potential of iPSC-ECs and indicate that paracrine signaling is the fundamental mechanism of the promising cardioprotective effects mediated by iPSC-ECs [7]. Exosomes are soluble factors released by the transplanted cells and may provide an alternative treatment approach for ischemic diseases [8, 9]. Therefore, it is interesting to investigate whether hiPSC-EC exosomes could become a novel acellular option to promote myocardial healing after MI and to elucidate the underlying mechanism.

Defects in Ca^2+^ homeostasis are a critical hallmark of a failing heart, as cardiomyocyte function depends on synchronized movements of Ca^**2+**^ cycling, which contributes to the heart rhythm and excitation–contraction coupling. There is cumulative evidence showing gross Ca^2+^ homeostasis disorders after MI, such as decreased Ca^2+^ transients, reduced sarcoplasmic reticulum (SR) Ca^2+^ content, and diminished SR Ca^2+^ uptake and sequestration. These abnormalities are attributed to the expression and function alterations of Ca^2+^-handling proteins and transporters. Ryanodine receptor 2 (RyR-2), which causes the release of Ca^2+^ from the SR, could be a potential therapeutic target in MI [10]. Numerous studies have raised the possibility that the dysfunction of RyR-2 induced by oxidative stress during HF could contribute to the reduction of SR content and Ca^2+^ transient because the channels cannot close during the diastole [11]. Moreover, SR Ca^2+^ ATPase 2a (SERCA-2a), which pumps Ca^2+^ back into the SR, and Na^+^/Ca^2+^ exchange 1 (NCX-1), which transports Ca^2+^ out of the cell, have synergistic or antithetic effects on removing Ca^2+^ from the cytosol [12, 13]. The functions of SERCA-2a and NCX-1 may be impaired because of inhibition of their expression or functional depression after MI, which might contribute to Ca^2+^ overload in the cytosol and cardiac dysfunction [12, 13]. Hence, modulating the Ca^2+^ homeostasis might be a potential therapeutic approach for MI. However, it is unclear whether and how hiPSC-EC exosomes could maintain intracellular Ca^2+^ homeostasis and promote cardiomyocyte survival to ameliorate infarcted heart function and reduce harmful remodeling. Moreover, considering that microRNAs are one of the most active components contained in exosomes [14–16], there is a strong rationale to elucidate which microRNA contained in the hiPSC-EC exosomes is the main factor that regulates the Ca^2+^ homeostasis and protects the cardiomyocytes from ischemic injury.

Therefore, in this study, we extracted the exosomes from hiPSC-ECs and identified their characteristics. Then, combining in vitro and in vivo strategies, we investigated (i) whether hiPSC-EC exosome administration could promote cardiomyocyte survival to repair the infarcted heart after MI; (ii) whether hiPSC-EC exosomes could modulate intracellular Ca^2+^ homeostasis to protect resident cardiomyocytes; (iii) which microRNA is the most abundant and mainly contributes to hiPSC-EC exosome-mediated beneficial effects; and (iv) the underlying mechanisms.

## Methods

### hiPSC-EC generation and characterization

hiPSCs used in this study were reprogrammed from human umbilical cord blood mononuclear cells via transduction of OCT4, SOX2, KLF4, and cMYC. Differentiation of endothelial cells was carried out as previously reported [17]. Then, hiPSC-ECs were enriched to > 95% by flow cytometry selection for both CD31 and CD144 expressions. The differentiated hiPSC-ECs were characterized via immunofluorescent staining of CD31, CD144, and von Willebrand factor (VWF); their purity was analyzed using flow cytometry. The Dil-conjugated acetylated low-density lipoprotein (Ac-LDL) uptake assay was also performed as described previously [18].

### Tube formation

Tube formation was evaluated as previously reported [18]. Briefly, Matrigel solution was placed in a 24-well tissue culture plate at 37ºC and allowed to solidify for 1 h. hiPSC-ECs (1.5 × 10^5^ cells/well) were seeded onto the solidified matrix with fresh EGM 2-MV medium (Lonza, CC-3202, Switzerland) in the presence of 50 ng/mL VEGF and incubated under standard cell culture conditions for 24 h; then, 2 μg/mL calcein was added to the wells and incubated with the cells for 20 min. The cells were viewed under a Zeiss inverted fluorescence microscope. The tube-like structures with lengths equal to at least four times their widths were counted, and tube formation was quantified as the mean number of tubes per field.

### hiPSC-EC exosome isolation and identification

hiPSC-EC-secreted exosomes (EC-Exo) were collected and purified from the culture medium as described previously [19, 20]. Briefly, 70–80% confluent hiPSC-ECs were washed with phosphate-buffered saline (PBS) and switched to a fresh exosome-depleted culture medium. After culturing for 48 h, the medium was collected and centrifuged at 1500 rpm for 10 min. The supernatant was passed through 0.22-μm filters to remove the cell debris. Finally, the supernatant was purified using an ExoQuick™ Precipitation Solution Kit (SBI, EXOQ5A-1, USA) to isolate the EC-Exo. The EC-Exo was resuspended in PBS and stored at − 80 °C for further experiments.

The particle size distributions and yield of exosomes reconstituted in PBS were determined using nanoparticle tracking analysis with NanoSight. The protein content was measured using the MicroBCA Protein Assay Kit (Beyotime, P0012, China), and the expression levels of exosomal marker proteins (ALG-2-interacting protein X [Alix], tumor susceptibility gene 101 protein [TSG101], CD63, and CD9) were evaluated using Western blot. The ultrastructure of exosomes was evaluated using transmission electron microscopy as described previously [6]. Congruously, miR-100-5p inhibitor (100 nM) oligonucleotides (RiboBio, miR10000098, China) or negative control (NC) oligonucleotides (RiboBio, miR2N0000001, China) were transfected into hiPSC-ECs using the Ribo FECT™ CP Transfection Kit (RiboBio, C10511-05, China). Then, exosomes secreted by the transfected hiPSC-ECs were isolated and identified as EC-Exo^anti−miR−100−5p^ or EC-Exo^NC^.

### Exosome uptake assessment

The hiPSCs were differentiated into cardiomyocytes (hiPSC-CMs) as previously reported [6]. To monitor the internalization of hiPSC-EC exosomes, the purified hiPSC-EC exosomes were labeled using a PKH26 Red Fluorescent Cell Linker Kit (Sigma-Aldrich, PKH26PCL-1KT, USA) for in vitro studies following the manufacturer’s instructions. Then, the prelabeled exosomes were added to hiPSC-CMs, which were subsequently fixed and stained for immunofluorescence.

### In vitro exosome cytoprotection assay

The hiPSC-CMs were seeded onto 24-well plates or four-chamber slides and separately treated with PBS, EC-Exo (1 μg/mL), EC-Exo^NC^ (1 μg/mL), EC-Exo^anti−miR−100−5p^ (1 μg/mL), mimic NC (100 nM), and miR-100-5p mimic (100 nM). Mimic NC and miR-100-5p mimic were transfected using the Lipofectamine RNAi_MAX_ Kit (Thermo Fisher Scientific, 13,778,075, USA). Then, cardiomyocytes were cultured under normal or oxygen–glucose deprivation conditions (glucose-free DMEM without serum, 1% O_2_/5% CO_2_/94% N_2_) for 48 h. Apoptosis was evaluated using an In Situ Cell Death Detection Kit (Roche Applied Science, 12,156,792,910, Germany). Lactate dehydrogenase (LDH) leakage in the culture medium was determined using a CytoTox-One homogenous membrane integrity assay (Promega, G7891, USA). The ATP content was measured in homogenized hiPSC-CMs using an ATP Bioluminescence Assay Kit (Sigma-Aldrich, 11,699,709,001, USA). The Ca^2+^ transients were evaluated by incubating the hiPSC-CMs with a Ca^2+^ indicator, Fura-2 AM (Beyotime, S1052, China), and electrically stimulating the cells. Then, the ratio of fluorescence emitted at 340 nm and 380 nm was recorded using a Ca^2+^-recording system (IonOptix, USA) [21].

### Cell migration

Cell migration assay was performed as previously described [22]. When human cardiac fibroblasts were grown to 100% confluent monolayers, the cells were scratched and washed three times with PBS. Then, EC-Exo (1 μg/mL) or PBS (equal volume to the exosomes) was added with the culture media into the 12-well plates. The marked areas were photographed using a Zeiss inverted microscope before and after different treatments for 24 h.

### Mice housing and husbandry

The 8–11-week-old male NOD-Prkdc^scid^ Il2rg^em1^/Smoc mice (M-NSG, 22–26 g) were purchased from Shanghai Model Organisms Center (Shanghai, China) and housed in the SPF environment. Mice were kept in individually ventilated cages at a temperature of 20˚C to 24˚C, humidity of 50% to 60%, and 60 air exchanges per hour. All mice were maintained on a regular diurnal lighting cycle (12:12 light/dark) with ad libitum access to food and water. Environmental enrichment included nesting material, PVC pipe, and shelter. All materials were autoclaved before use, including individually ventilated cages, lids, feeders, bottles, bedding, and water.

### Murine model of MI

In our study, we used the double-blinding method. On the one hand, mice were randomly divided into different groups (using computer-generated random numbers); on the other hand, the investigators were blinded to group allocation and assessed the outcome after different groups were treated. For Inclusion/exclusion criteria, mice (8–11 weeks old, male) with normal and similar weight (22–26 g), appearance, and hair were included in the study. Before the operation, the baseline cardiac function of each mouse was evaluated by echocardiography, and mice with LV ejection fraction (LVEF) below 50% were excluded from the study. During the operation, we included the mice only when the ligation of left anterior descending (LAD) was successful, which was defined by the pale anterior wall and ST elevation observation on ECG.

The individual mouse was considered the experimental unit within the studies, and the method of resource equation approach was used to determine the sample size in this study as described previously [23]. Seventy-eight mice were simultaneously randomized to the sham group, MI group, and MI + EC-Exo group, of which three mice did not meet our inclusion and exclusion criteria because no ST elevation was observed on one animal, and two animals postoperatively died (autopsy unable to identify the cause of death). Therefore, a total of 75 animals were included in the analysis of this study. MI was produced in mice by permanently ligating the LAD coronary artery [19]. All surgeries were operated by the same surgeon. Briefly, the mice were placed in an induction chamber; they received 2% isoflurane and were then connected to a small rodent ventilator. Afterward, the chest was opened to expose the heart, left auricle, and LAD. The LAD was permanently ligated with an 8–0 nylon suture. Animals in the MI + EC-Exo group (*n* = 27) received hiPSC-EC exosomes (20 μg in 20 μL of PBS) by intramyocardial injection to three sites in the anterior wall of the left ventricle (LV); animals in the MI group (*n* = 24) received 20 μL of PBS by intramyocardial injection; and animals in the sham group (*n* = 24) underwent all surgical procedures for MI induction, except for occlusion, and recovered without any of the experimental treatments. The animal’s temperature was maintained during surgery by using a heating pad. The convalescent mice were placed in a separate cage to avoid injury by the unoperated mice. Finally, the mice received intraperitoneal injections of buprenorphine (0.1 mg/kg) every 12 h for up to 3 days and intraperitoneal injections of carprofen (5 mg/kg) every 12 h for up to one day after the surgery for pain management. At the end of the experiment (3, 7, or 28 days after MI or sham operation), the hearts were collected and cut in half; one half was used for histological analyses, while the other half was used for protein extraction.

### Echocardiography

The primary outcome of this study will be cardiac function assessed by echocardiography using a Vevo 2100 Imaging System (Visual Sonics Inc., Canada) as described previously [19, 22]. Briefly, the mice were lightly anesthetized with 2% isoflurane until the heart rate stabilized at 400–500 bpm; then, both conventional two-dimensional mode and M-mode images of the heart were acquired in a parasternal short-axis view. Vevo Analysis software was used to calculate the LVEF and LV fractional shortening (LVFS).

### Hemodynamic measurement

LV pressures were continuously monitored using a PowerLab system (AD Instrument, Australia) on day 28 after MI as described previously [24]. The LV developed pressure (LVDP), LV end-diastolic pressure (LVEDP), LV maximum ascending rate of pressure (+ dp/dt_max_), and LV maximum declining rate of pressure (− dp/dt_max_) were assessed.

### Electrocardiogram monitoring

Electrocardiograms of MI mice were acquired as previously described [25]. Briefly, the mice were placed in a chamber and anesthetized with 2% isoflurane on 7 days after MI. Then, they were fixed on the operating table and connected to a small rodent ventilator for subsequent electrocardiogram monitoring. Arrhythmias were provoked by a double intraperitoneal injection of 0.1 mg/kg isoproterenol (Sigma-Aldrich, 1,351,005, USA) at a 30-min interval. Electrocardiograms were continuously recorded using a PowerLab system for 3 h. The number of arrhythmic events, such as atrioventricular block, premature ventricular contraction, ventricular tachycardia, or ventricular fibrillation, was counted for 1 min every 10 min.

### 2,3,5-Triphenyl tetrazolium chloride (TTC) staining

Mouse hearts were collected 3 days after MI and cut into 4-mm slices perpendicular to the LAD from the apex to the base. Then, slices were immersed in 1% TTC (Sigma-Aldrich, T8877, USA) solution (pH 7.4) at 37 °C in the dark for 20 min to visualize the infarct size.

### Masson’s trichrome staining

On day 28 after MI, the hearts were harvested, frozen, and cut into 8-μm-thick sections; then, the sections were stained with a modified Masson's Trichrome Stain Kit (Solarbio, G1346, China). Infarct size was calculated as the total scar area divided by the LV area, as previously reported [22, 24].

### Immunofluorescence

Immunofluorescence staining was performed for the cells or heart tissue sections as previously described [26]. For the fibroblast proliferation assay, fibroblasts were immunofluorescently stained with vimentin and Ki67 antibodies. For wheat germ agglutinin (WGA) staining, the sections were stained using a FITC-labeled WGA dye (Sigma-Aldrich, L4895, USA). For terminal deoxynucleotidyl transferase dUTP nick end labeling (TUNEL) staining, the sections were stained with an In Situ Cell Death Detection Kit. Cardiomyocytes were identified by the expression of cardiac troponin I (cTnI), and nuclei were counterstained with 4′,6-diamidino-2-phenylindole (DAPI).

Pictures are realized with the TCS SP8 STED 3X (Leica, German), DM1000 (Leica), FLUOVIEW FV3000 (OLYMPUS, Japan), or IX83 (OLYMPUS) microscope and analyzed with Leica Application Suite X or OLYMPUS OlyVIA software. For the images of CD31, CD144, VWF, calcein, and Ac-LDL staining, the zoom is 400 × with a resolution of 0.25 μm/pixel; for the images PKH26/α-actinin and TUNEL/cTnI staining in hiPSC-CMs, the zoom is 600 × with a resolution of 0.21 μm/pixel; for the images of WGA/cTnI and TUNEL/cTnI staining in heart tissue slices, the zoom is 400 × with a resolution of 0.38 μm/pixel; and for the images of vimentin/Ki67 staining in fibroblasts, the zoom is 200 × with a resolution of 0.46 μm/pixel. All images of the same study were processed with the same parameters.

### Simultaneous measurement of cell shortening and Ca^2+^ transients

Four weeks after MI or sham operation, LV cardiomyocytes were isolated from the hearts using a standard enzymatic method as described previously [21]. The collected cardiomyocytes were incubated with a Ca^2+^ indicator (Fura-2 AM) and stimulated electrically using square-wave pulses at 0.5, 1, and 2 Hz. Ca^2+^ transients and cell shortening were detected simultaneously using a video-based motion edge detection and Ca^2+^-recording system (IonOptix) as previously described [21]. Caffeine (10 mmol/L) was applied into normal Tyrode’s solution (NT) or the Na^+^-free and Ca^2+^-free (0 Na^+^/0 Ca^2+^) Tyrode’s solution. The amplitude and maximum upstroke velocity (V_max_) of caffeine-induced Ca^2+^ transients, the rate constants of SERCA-mediated Ca^2+^ transport, and the rate constants of NCX-mediated Ca^2+^ transport were determined as previously described [27].

### Ca^2+^-ATPase activity measurement in cardiac SR

Cardiac SR samples were prepared as described previously [28]. Then, SR Ca^2+^-ATPase activities were determined by measuring the inorganic phosphate (Pi) liberated from ATP hydrolysis and using a Ca^2+^-pump ATPase Enzyme Assay Kit (Jiancheng Bioengineering, A070-4-2, China).

### Western blot

Protein extracts were measured using sodium dodecyl sulfate–polyacrylamide gel electrophoresis (SDS–PAGE) gels in a Protein Electrophoresis Apparatus (Bio-Rad, USA) and then transferred to a polyvinylidene difluoride membrane, as reported previously [21]. The membrane was incubated overnight with the primary antibodies against RyR-2, SERCA-2a, NCX-1, PLB, p-Ser^16^-PLB, p-Thr^17^-PLB, and protein phosphatase 1*β* (PP-1*β*). Glyceraldehyde-3-phosphate dehydrogenase (GAPDH) or *β*-tubulin antibody was labeled as a control for unequal loading. Then, the membranes were incubated with horseradish peroxidase (HRP)-conjugated secondary antibodies at room temperature and exposed via enhanced chemiluminescence.

### Next-generation sequencing (NGS)

microRNA sequencing and data analysis of hiPSC-EC exosome samples were executed by LC Sciences (China). The total exosomal RNA was purified using a miRcute kit (TIANGEN, China, DP501), and the RNA quality and quantity were validated using a 2100 Bioanalyzer (Agilent). Following the TruSeq Small RNA Sample Preparation protocol, sequencing libraries were generated by ligating specific RNA adapters to the 5′ and 3′ ends for each sample, followed by reverse transcription, amplification, and purification of small-RNA libraries targeting the size range of 22 to 30 nucleotides. Sequencing was performed on the Illumina HiSeq 2500 platform, and a customized in-house program, ACGT101-miR v4.2 (LC Sciences), was used to remove adapter dimers, junk, low complexity, common RNA families, and repeats from the raw reads. Unique sequences with a length of 18 to 26 nucleotides were mapped to specific species precursors in miRBase 22.0 using a BLAST search to identify known miRNAs and novel 5p- and 3p-derived miRNAs [29, 30].

### Luciferase reporter assay

The PP-1*β* sequences containing 127–133 bp were amplified and cloned in the pmirGLO dual-luciferase microRNA target expression vector (Promega, E1330, USA). The mutant PP-1*β* 3′UTR pmirGLO vector was generated using QuikChange II XL Site-Directed Mutagenesis Kit (Stratagene, 200,522, USA) according to the manufacturer’s protocol. Next, 293 T cells were co-transfected with miR-100-5p mimic and either the PP-1*β* 3′UTR pmirGLO vector or the mutant PP-1*β* 3′UTR pmirGLO vector. Then, the luciferase activity of the cells was analyzed using a Dual-Glo Luciferase Assay System (Promega, E2920, USA). The relative luciferase activity was determined based on the ratio of firefly/Renilla luminescence.

### RNA interference

The hiPSC-CMs were cultured and transfected with scramble control and PP-1*β* siRNA (100 nM, RiboBio) using Lipofectamine RNAi_MAX_ (Thermo Fisher Scientific, 13,778,075, USA) in line with the manufacturer’s protocols.

### RNA isolation and real-time quantitative polymerase chain reaction (RT-qPCR)

The microRNA was extracted from EC-Exo as well as fibroblast-secreted exosomes, which were always used as a negative control in exosome-associated therapies [20, 31], using the TRIzol reagent (Thermo Fisher Scientific, 15,596,018, USA) according to the manufacturer’s protocols. The microRNAs were reverse-transcribed using a TaqMan microRNA Reverse Transcription Kit (ABI, 4,366,596, USA) and analyzed using BeyoFast™ SYBR Green qPCR Mix (Beyotime, D7260, China) on ABI-7900 Real-Time PCR Detection System. The relative expression of microRNAs was normalized to that of U6*.* The relative expression level was calculated using the 2^−ΔΔCt^ method.

### Statistical analysis

Data are presented as mean ± standard error of mean (SEM). Firstly, the normal distribution of all data was assessed by using Shapiro–Wilk normality test. Then, differences between two mean values were evaluated via Student’s *t*-test, while analysis of variance (ANOVA) with the Tukey’s post hoc test was used for multiple comparisons. All statistical analyses were performed using Statistical Product and Service Solutions (SPSS) software (version 18.0; IBM Corp., Armonk, NY, USA). *P* < 0.05 was considered statistically significant.

## Results

### Characterization of hiPSC-ECs and hiPSC-EC exosomes

**Fig. 1:**
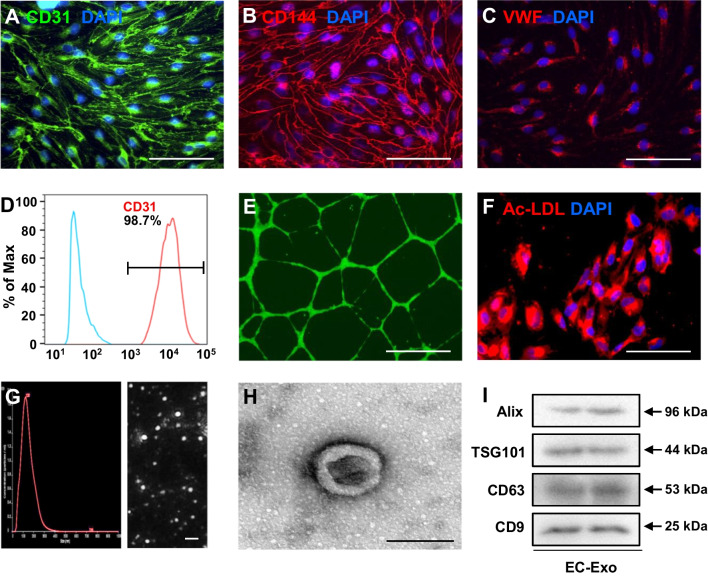
Characterization of hiPSC-ECs and exosomes secreted from hiPSC-ECs. **A–C** hiPSCs were differentiated into endothelial cells (hiPSC-ECs). hiPSC-ECs were characterized via immunofluorescent analyses of the expressions of **A** CD31, **B** CD144, and **C** von Willebrand factor (VWF). Nuclei were counterstained with 4′,6-diamidino-2-phenylindole (DAPI) (bar = 100 μm). **D** The purity of hiPSC-ECs was determined by FACS analysis of CD31. **E** hiPSC-ECs were suspended in a culture medium supplemented with 50 ng/mL vascular endothelial growth factor (VEGF) and then plated on Matrigel; 24 h later, the cells were labeled with calcein, and tube formation was evaluated under a light microscope (bar = 100 μm). **F** The uptake of Dil-conjugated acetylated low-density lipoprotein (Ac-LDL) was evaluated in hiPSC-ECs; nuclei were counterstained with DAPI (bar = 100 μm). **G–I** Exosomes were isolated from the culture medium of hiPSC-ECs; then, **G** exosome size was evaluated via nanoparticle tracking analysis (bar = 500 nm), **H** exosome morphology was evaluated via electron microscopy (bar = 100 nm), and **I** the presence of exosome marker proteins (ALG-2-interacting protein X [Alix], tumor susceptibility gene 101 protein [TSG101], CD63, and CD9) was evaluated via Western blot. Full-length blots are presented in Additional file 1: Fig. S1

The hiPSCs were reprogrammed from human umbilical cord blood mononuclear cells and differentiated into hiPSC-ECs via established protocols [17]. Immunofluorescence staining assessments showed that hiPSC-ECs expressed EC-specific (CD31, CD144, and VWF) markers (Fig. 1A–C), and fluorescence-activated cell sorting (FACS) of CD31 demonstrated that the achieved purity of hiPSC-ECs was more than 98% (Fig. 1D). The hiPSC-ECs also exhibited EC properties, including the formation of tube-like structures on Matrigel in the presence of vascular endothelial growth factor (VEGF) (Fig. 1E) and uptake of Ac-LDL (Fig. 1F). Subsequently, exosomes were extracted from the hiPSC-EC culture medium via centrifuge ultrafiltration and precipitation in polyethylene glycol. Nanoparticle tracking (Fig. 1G) and transmission electron microscopy (Fig. 1H) analyses indicated that the isolated exosomes were almost 100 nm in diameter and had a double-membrane-bound, cup-shaped typical shape. Congruously, Western blot confirmed that hiPSC-EC exosomes expressed the exosome markers, including Alix, TSG101, CD63, and CD9 (Fig. 1I, Additional file 1: Fig. S1). Taken together, these results demonstrate that the generated hiPSC-ECs and isolated hiPSC-EC exosomes exhibited typical characteristics.

### hiPSC-EC exosomes protect cardiomyocytes from oxygen and glucose deprivation (OGD) injury and promote EC tube formation in vitro

**Fig. 2:**
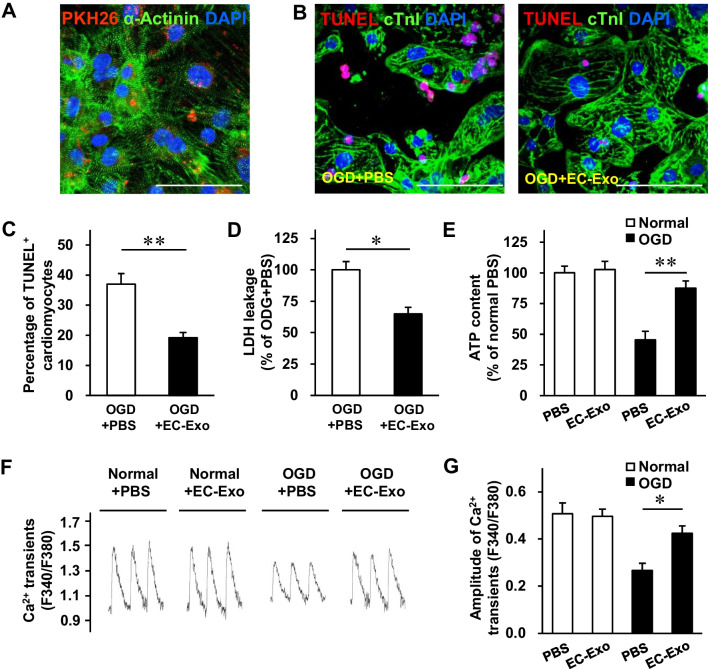
In vitro cytoprotective effects of hiPSC-EC exosomes on hiPSC-CMs. **A** hiPSC-derived cardiomyocytes (hiPSC-CMs) were incubated for 24 h with PKH26-labeled hiPSC-EC exosomes (EC-Exo); then, the cardiomyocytes were fixed and immunofluorescently stained for α-actinin, and nuclei were counterstained with DAPI. Exosomes that had been taken up by the cardiomyocytes were identified using PKH26 fluorescence (bar = 100 μm). **B–G** hiPSC-CMs were cultured under normal or oxygen and glucose deprivation (OGD) conditions with PBS or hiPSC-EC exosome treatment for 48 h. **B** hiPSC-CMs were fixed, immunofluorescently stained for cardiac troponin I (cTnI) expression, and stained by terminal deoxynucleotidyl transferase dUTP nick end labeling (TUNEL); then, nuclei were counterstained with DAPI (bar = 100 μm). **C** Quantification for TUNEL^+^ cardiomyocytes. **D** The activity of lactate dehydrogenase (LDH) released in the culture media was measured using a LDH release assay kit. **E** hiPSC-CM ATP content was analyzed using an ATP bioluminescence assay kit. **F–G** hiPSC-CMs were incubated with a Ca^2+^ indicator (Fura-2 AM); then, Ca^2+^ transients were recorded with continuous 0.5 Hz electric stimulation. **F** The representative traces of hiPSC-CMs are shown. **G** Quantification of Ca^2+^ transient amplitudes. Quantitative data are presented as mean ± SEM. *n* = 4 independent experiments in **C**, **D**, and **E** and *n* = 5 independent experiments in **G**. Significance was evaluated via Student’s *t*-test in (**C** and **D**) and one-way ANOVA followed by Tukey’s post hoc test in (**E** and **G**). *p < 0.05 and **p < 0.01

We examined whether hiPSC-EC exosomes possess a protective effect against OGD injury in cardiomyocytes in vitro. Images of PKH26 fluorescence indicated that when hiPSC-CMs were cultured with PKH26-labeled hiPSC-EC exosomes for 24 h, numerous exosomes were taken up by the cardiomyocytes (Fig. 2A). Next, hiPSC-CMs were cultured under normal or OGD conditions and treated with PBS or hiPSC-EC exosomes for succedent analyses. TUNEL staining demonstrated that apoptosis of cardiomyocytes with hiPSC-EC exosome treatment was notably lower than that with PBS treatment under OGD injury (Fig. 2B, C). There were no apparent differences between the two treatments under normal conditions (Additional file 1: Fig. S2). In line with this, hiPSC-EC exosome treatment markedly alleviated LDH leakage (Fig. 2D) and reversed the ATP content of cardiomyocytes (Fig. 2E) under OGD injury. Furthermore, the amplitude of Ca^2+^ transients (F340/F380) of OGD cardiomyocytes after hiPSC-EC exosome treatment was significantly higher than that after PBS treatment (Fig. 2F, G). Taken together, the hiPSC-EC exosomes exhibited the potential to protect cardiomyocytes from OGD injury by impeding apoptosis, promoting cell survival, improving energy metabolism, and maintaining intracellular Ca^2+^ homeostasis*.*

Moreover, we explored whether the hiPSC-EC exosomes could target other cardiac cells (e.g., fibroblasts and ECs) in vitro. The results demonstrated that hiPSC-EC exosomes had no significant impact on fibroblast migration (Additional file 1: Fig. S3A, B) and proliferation (Additional file 1: Fig. S3C, D) under normal and TGF-*β*1-treated conditions. However, the EC tube-forming activity (Additional file 1: Fig. S3E, F) was markedly enhanced after hiPSC-EC exosome treatment. These findings demonstrate that hiPSC-EC exosomes also act directly on ECs to promote angiogenesis.

### hiPSC-EC exosomes improve cardiac function and limit adverse remodeling with no increase in the risk of arrhythmia in a murine MI model

**Fig. 3:**
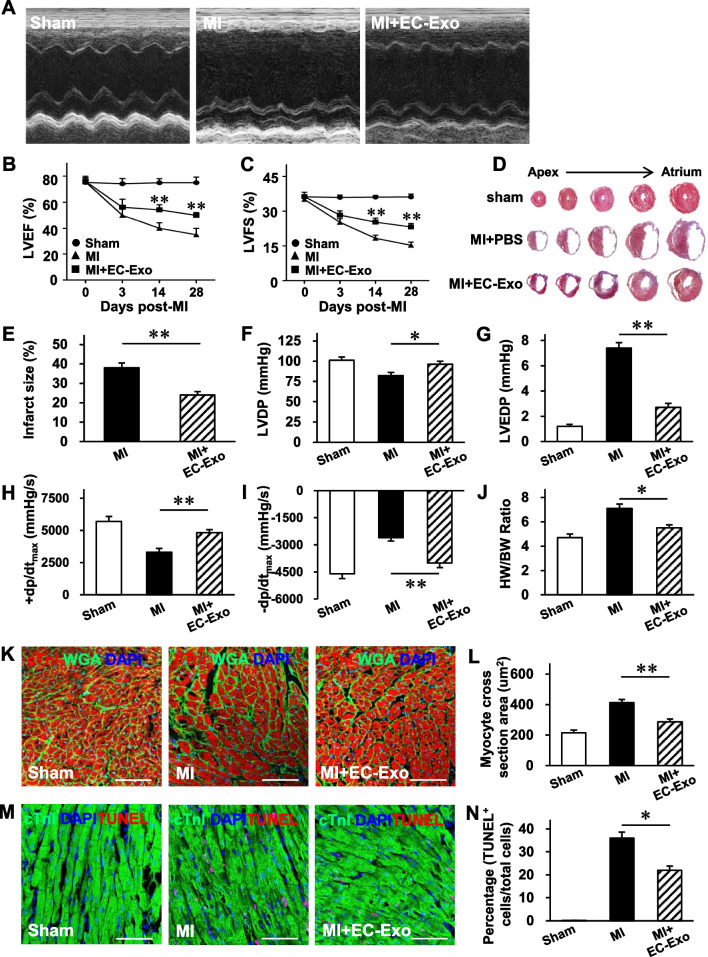
hiPSC-EC exosomes promote the recovery of cardiac function and limit heart remodeling in a mouse model of myocardial infarction (MI). The mice were divided into the sham, MI, and MI + EC-Exo groups. **A–C** Cardiac function was evaluated on days 3, 14, and 28 after MI or sham surgery via **A** echocardiographic assessments. Quantification of **B** left ventricular (LV) ejection fraction (LVEF) and **C** LV fractional shorting (LVFS). **D** Representative images of infarct size after 28 days with Masson trichrome staining. **E** Quantitative data of infarct size (%) were calculated by infarct areas/total LV areas. **F–I** Hemodynamic measurements of **F** LV developed pressure (LVDP), **G** LV end-diastolic pressure (LVEDP), **H** LV maximum ascending rate of pressure (+ dp/dt_max_), and **I** LV maximum declining rate of pressure (− dp/dt_max_) were performed 28 days post-MI. **J** Heart-to-body weight ratio (HW/BW) 28 days after MI. **K** Sections from the border zone (BZ) of the infarcted heart (28 days after MI) were stained with wheat germ agglutinin (WGA) to identify the cellular borders and with cTnI to visualize CMs; nuclei were counterstained with DAPI (bar = 100 μm); then, **L** cardiomyocyte cross-sectional surface areas were measured. **M** The sections obtained from the BZ (3 days post-MI) were stained with antibodies against cTnI, apoptotic cells were identified via a TUNEL staining, and nuclei were counterstained with DAPI (bar = 100 μm). **N** Apoptosis was quantified as the percentage of TUNEL^+^ cells. Quantitative data are presented as mean ± SEM. *n* = 9 in sham group, *n* = 9 in MI group, and *n* = 11 in MI + EC-Exo group (**B**, **C**, and **F**–**J**); *n* = 5 in sham group, *n* = 5 in MI group, and *n* = 6 in MI + EC-Exo group (**E**, **L**, and **N**). Significance was evaluated via Student’s *t*-test in (**E**) and one-way ANOVA followed by Tukey’s post hoc test in (**B**, **C**, **F**–**J**, **L**, and **N**). *p < 0.05 and **p < 0.01 vs. MI group or as indicated

We investigated the therapeutic efficacy of hiPSC-EC exosomes against MI injury in mice. Cardiac function was evaluated via echocardiographic assessments 3, 14, and 28 days after MI or sham surgery (Fig. 3A). The LVEF and LVFS in both MI and MI + EC-Exo groups declined 3 days after MI without a significant difference between the groups (Fig. 3B, C). However, these parameters were further worsened in the MI group, while hiPSC-EC exosome treatment could significantly reverse the deteriorations in heart function 14 and 28 days after the MI (Fig. 3B, C). Cardiac infarct size was evaluated by TTC staining 3 days after MI and Masson’s trichrome staining 28 days after MI. The histological analysis revealed that the infarct size was notably attenuated in the MI + EC-Exo group compared to the MI group (Fig. 3D, E, Additional file 1: Fig. S4). In line with this, the hemodynamic measurements demonstrated that LVDP (Fig. 3F), LVEDP (Fig. 3G), + dp/dt_max_ (Fig. 3H), and -dp/dt_max_ (Fig. 3I) were significantly improved in the MI + EC-Exo animals compared to the MI animals. Furthermore, the apparent adverse LV remodeling was progressively reversed after hiPSC-EC exosome treatment 28 days post-MI, as evidenced by a reduction of the ratio of heart weight to body weight (Fig. 3J) and cardiomyocyte size detected by WGA staining (Fig. 3K, L). In addition, TUNEL staining analysis demonstrated that TUNEL^+^ cardiomyocytes in the border zone of the infarcted hearts were significantly eliminated in MI + EC-Exo animals compared to the MI animals three days after MI (Fig. 3M, N). Taken together, these results demonstrate that hiPSC-EC exosome administration not only ameliorated the deterioration of heart function but also attenuated the harmful LV remodeling after MI.

Increasing evidence shows that the transplantation of pluripotent stem cell-derived cardiomyocytes predisposes recipients to arrhythmias [32]. Therefore, an arrhythmia risk assessment was performed after hiPSC-EC exosome transplantation in MI mice. No apparent differences in arrhythmia episodes induced by isoproterenol, including atrioventricular block, premature ventricular contraction, ventricular tachycardia, and ventricular fibrillation, were observed between the MI and MI + EC-Exo groups on day 7 after MI (Additional file 1: Fig. S5). These results demonstrated that hiPSC-EC exosome treatment is a safe and feasible therapeutic strategy for MI without increasing arrhythmia risk.

### hiPSC-EC exosomes improve cardiomyocyte contraction and Ca^2+^ homeostasis after MI

**Fig. 4:**
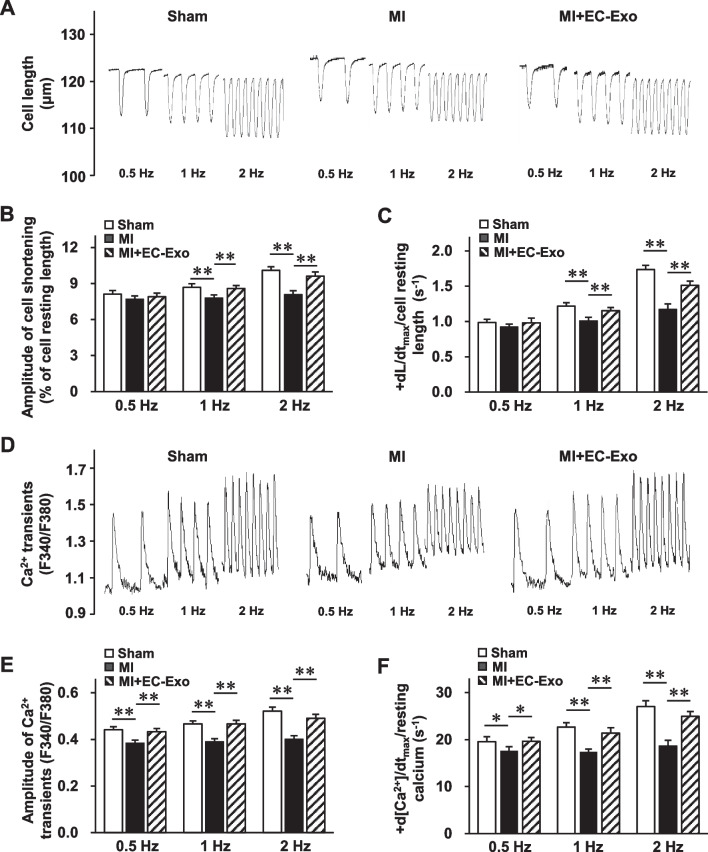
Cell shortening and calcium transient assessments in isolated cardiomyocytes after MI. The rod-shaped cardiomyocytes were isolated from mouse left ventricles of the sham group, MI group, and MI + EC-Exo group on day 28 after MI or sham surgery. Cardiomyocytes were incubated with a Ca^2+^ indicator (Fura-2 AM); then, cell shortening and Ca^2+^ transients were recorded under continuous 0.5, 1, and 2 Hz electrical stimulation. **A** Representative traces of cell shortening. Quantification of **B** amplitude of cell shortening/cell resting length and **C** maximum upstroke velocity of cell shortening (+ dL/dt_max_)/cell resting length. **D** Representative traces of calcium transients. Quantification of **E** amplitude of Ca^2+^ transient and **F** maximal ascending rate in cell contractile Ca^2+^ transients (+ d[Ca^2+^]/dt_max_)/resting calcium. Quantitative data are presented as mean ± SEM. A total of 40–44 cardiomyocytes from four different hearts were measured in each group. Significance was evaluated via one-way ANOVA followed by Tukey’s post hoc test in (**B**, **C**, **E**, and **F**). **p < 0.01

It is well established that the disorder of intracellular Ca^2+^ homeostasis, which is characterized by a dramatic decline in Ca^2+^ transients, reduced SR Ca^2+^ content, and impaired Ca^2+^ uptake and extrusion, is one of the most severe adverse effects of failing hearts [33]. Thus, we further examined whether hiPSC-EC exosome treatment could rescue these abnormalities. Compared to the sham group, LV cardiomyocytes isolated from animals of the MI group showed a significant decrease in the ratio of cell shortening amplitude to cell resting length at 1 and 2 Hz. However, this decrease was dramatically attenuated in the MI + EC-Exo group (Fig. 4A, B). Similar results were observed in the ratio of maximum upstroke velocity of cell shortening (+ dL/dt_max_) to cell resting length after hiPSC-EC exosome treatment (Fig. 4C). In line with this, the amplitude of Ca^2+^ transients was notably impaired in the MI group at 0.5, 1, and 2 Hz stimulation, while these abnormalities were markedly alleviated in the MI + EC-Exo group (Fig. 4D, E), which is concordant with the ratio of maximal ascending rate of Ca^2+^ transients (+ d[Ca^2+^]/dt_max_) to resting Ca^2+^ transients (Fig. 4F). Taken together, these results demonstrate that hiPSC-EC exosomes dramatically improved the cardiomyocyte contraction and modulated the intracellular Ca^2+^ homeostasis in the infarcted hearts.

### Effects of hiPSC-EC exosomes on the function of Ca^2+^-handling proteins in infarcted hearts

**Fig. 5:**
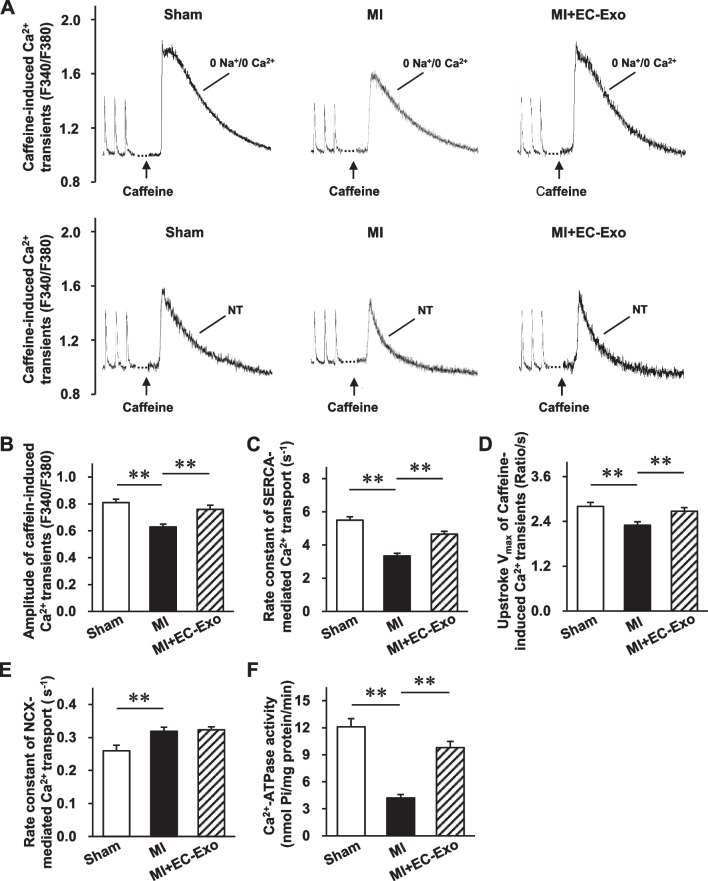
Sarcoplasmic reticulum (SR) Ca^2+^ content and Ca^2+^ transport rate assessments in isolated cardiomyocytes after MI. Ca^2+^ transients were evoked by 10 mM caffeine in ventricular myocytes from the sham group, MI group, and MI + EC-Exo group on day 28 after MI or sham surgery. **A** Representative traces of Ca^2+^ transient during caffeine-induced contraction in 0 Na^+^/0 Ca^2+^ Tyrode’s solution or normal Na^+^/Ca^2+^ Tyrode’s solution (NT). **B** Quantification of the amplitude of caffeine-induced Ca^2+^ transients in 0 Na^+^/0 Ca^2+^ Tyrode’s solution. **C** Rate constant of SERCA-mediated Ca^2+^ transport (the difference between the rate constant of caffeine-evoked Ca^2+^ transients and electric stimulation-evoked Ca^2+^ transients in NT solution). **D** Maximum upstroke velocity (V_max_) of caffeine-induced Ca^2+^ transient in 0 Na^+^/0 Ca^2+^ Tyrode’s solution. **E** Rate constant of Na^+^/Ca^2+^ exchanger-1 (NCX-1)-mediated Ca^2+^ transport (the difference between delay rate constant of caffeine-evoked Ca^2+^ transients in NT solution and in 0 Na^+^/0 Ca^2+^ solution). **F** SERCA activity was accessed using a Ca^2+^-pump ATPase enzyme assay kit. Quantitative data are presented as mean ± SEM. A total of 40–44 cardiomyocytes from four different hearts were measured in each group. Significance was evaluated via one-way ANOVA followed by Tukey’s post hoc test in (**B**–**F**). **p < 0.01

Because the disorders of intracellular Ca^2+^ homeostasis are mainly attributed to the function of critical Ca^2+^-handling proteins [12, 33–35], we examined caffeine-induced Ca^2+^ transients from isolated cardiomyocytes after MI to indirectly evaluate the function of main Ca^2+^-handling proteins (Fig. 5A). The markedly reduced caffeine-induced Ca^2+^ transient amplitude with 0 Na^+^/0 Ca^2+^ Tyrode’s solution in the MI group was notably reversed in the MI + EC-Exo group, indicating that hiPSC-EC exosomes enhanced the SR Ca^2+^ content (Fig. 5B). Subsequently, the rate constant of Ca^2+^ transport by SERCA, which indirectly represents the SERCA function, presented a striking reversal in the MI + EC-Exo group compared to the MI group (Fig. 5C). In addition, the V_max_ of caffeine-induced Ca^2+^ transients in 0 Na^+^/0 Ca^2+^ Tyrode’s solution dramatically declined in the MI group, while visible augmentation was confirmed in the MI + EC-Exo group, thereby revealing that hiPSC-EC exosomes could alter the RyR functional abnormality (Fig. 5D). However, the rate constant of Ca^2+^ transport by NCX, which indirectly represents the NCX function, showed no apparent differences between the MI + EC-Exo group and the MI group, while it was dramatically increased after MI (Fig. 5E). Similarly, the measurement of Ca^2+^-ATPase activity in the SR vesicles obtained from the LV tissue revealed that the dramatically declined Ca^2+^-ATPase activity in the MI group was notably reversed in the MI + EC-Exo group, which indicated the improvement of SERCA function after hiPSC-EC exosome treatment (Fig. 5F).

**Fig. 6:**
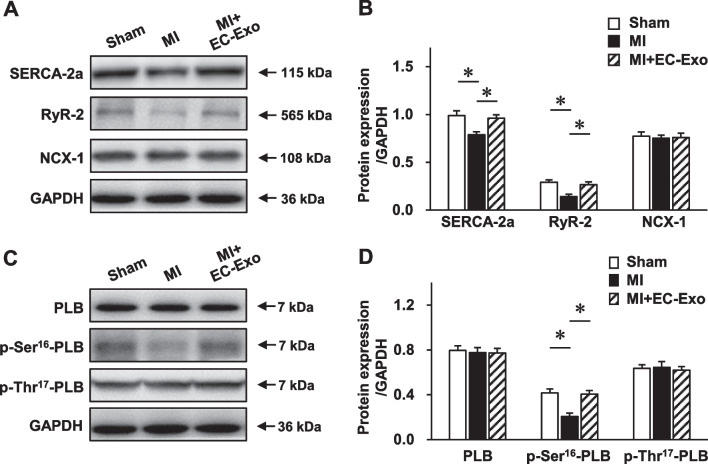
Evaluation of calcium-handling protein expression. Protein samples of heart tissues were collected from different groups 28 days post-MI or sham surgery. **A–B** Western blot to assess the expression levels of calcium-handling proteins, including ryanodine receptor 2 (RyR-2), NCX-1, and SERCA-2a. **A** Representative immunoblots. Full-length blots are presented in Additional file 1: Fig. S6. **B** Quantitative analysis of protein expression. **C–D** Western blot to assess the protein expression levels of phospholamban (PLB), p-Ser^16^-PLB, and p-Thr^17^-PLB. **C** Representative immunoblots. Full-length blots are presented in Additional file 1: Fig. S7. **D** Quantitative analysis of protein expression levels. Quantitative data are presented as mean ± SEM, *n* = 4 per group. Significance was evaluated via one-way ANOVA followed by Tukey’s post hoc test in (**B** and **D**). *p < 0.05

Furthermore, we performed Western blot to investigate the influence of hiPSC-EC exosomes on the expression of main Ca^2+^-handling proteins (Fig. 6A). The results revealed that the expression levels of SERCA-2a and RyR-2 were significantly downregulated in the MI group, while these detrimental abnormalities were dramatically diminished in the MI + EC-Exo group (Fig. 6A, B, Additional file 1: Fig. S6). However, no significant difference in NCX-1 protein expression was detected between the MI group and the MI + EC-Exo group (Fig. 6B, Additional file 1: Fig. S6). Subsequently, we evaluated the expression and phosphorylation levels of PLB (Fig. 6C, Additional file 1: Fig. S7), which is a major regulator of SERCA-2a [36]. According to the Western blot results, the protein expressions of PLB and p-thr^17^-PLB did not show any differences between the MI group and the MI + EC-Exo group (Fig. 6D, Additional file 1: Fig. S7), while the markedly decreased p-Ser^16^-PLB in the MI group was significantly reversed in the MI + EC-Exo group (Fig. 6D, Additional file 1: Fig. S7). Taken together, these results demonstrate that hiPSC-EC exosomes were not only able to enhance the protein expressions of SERCA-2a, RyR-2, and p-Ser^16^-PLB but also rescued the function of SERCA and RyR to maintain cardiomyocyte Ca^2+^ homeostasis after MI.

### hiPSC-EC exosome-contained miR-100-5p enhances the phosphorylation of PLB by targeting PP-1β

**Fig. 7:**
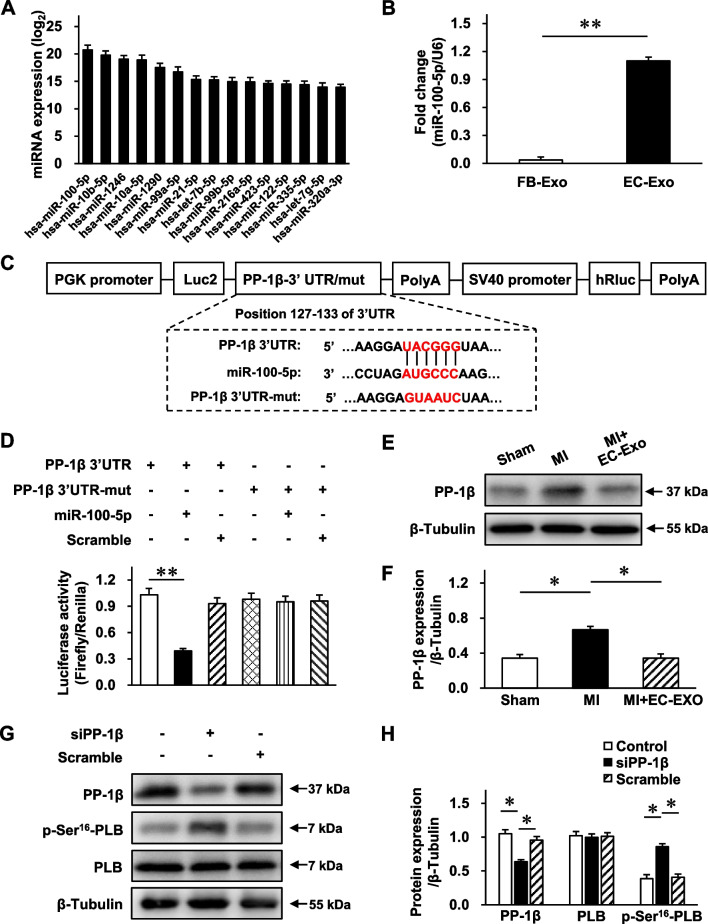
PP-1*β* is the direct target of miR-100-5p. **A** Total RNA was extracted from hiPSC-EC exosomes, and microRNA sequencing was performed. The expression of the 15 microRNAs that were most abundant is displayed in a bar graph; data are expressed as log_2_ of exosomal microRNA normalized read counts. **B** microRNA quantity of miR-100-5p in human dermal fibroblast exosomes (FB-Exo) and hiPSC-EC exosomes (EC-Exo) was measured via RT-qPCR and normalized to measurements in hiPSC-EC exosomes. **C–D** Luciferase activity analysis to investigate the miR-100-5p target gene, serine/threonine specific protein phosphatase 1 catalytic subunits *β* (PP-1*β*). **C** Wild-type or mutant dual-luciferase reporter plasmid was constructed according to the predicted binding sequence in 3′ UTR of PP-1*β* or mutant sequence; then, **D** the activities of Renilla and firefly luciferase were determined using the dual-luciferase reporter assay system. **E–F** Western blot was performed to assess the PP-1*β* protein expression in heart tissues 28 days post-MI or sham surgery. **E** Representative immunoblots. Full-length blots are presented in Additional file 1: Fig. S8. **F** Quantitative protein expression. **G–H** siPP-1*β* and scramble control were transfected into hiPSC-CMs to confirm gene-silencing efficiency and downstream gene regulation. **G** Representative immunoblots. Full-length blots are presented in Additional file 1: Fig. S9. **H** Quantitative protein expression. Quantitative data are presented as mean ± SEM. *n* = 4 independent experiments. Significance was evaluated via one-way ANOVA followed by Tukey’s post hoc test in (**D**, **F**, and **H**) and Student’s *t*-test in (**B**).  *p < 0.05 and **p < 0.01

MicroRNAs are the dominant active ingredients in exosomes and play a key role in cell communication [15, 16, 37]. Therefore, we extracted total RNA from hiPSC-EC exosomes and performed microRNA sequencing. Fifteen microRNAs that were most abundant in hiPSC-EC exosomes were presented (Fig. 7A). Considering that miR-100-5p was the most abundant microRNA in hiPSC-EC exosomes and that its content was further confirmed by RT-qPCR (Fig. 7B), the effect and mechanism of miR-100-5p were further investigated. Using TargetScan software, we predicted the potential target genes of miR-100-5p and selected PP-1*β* as a candidate target gene. PP-1*β* is one of the catalytic subunits of PP-1, which is the major isotype of serine/threonine phosphatase in cardiomyocytes and plays a pivotal role in regulating the phosphorylation of PLB at the Ser^16^ site [38]. Accordingly, a luciferase experiment was performed to verify the interaction between PP-1*β* and miR-100-5p using a dual-luciferase reporter plasmid containing wild-type (WT) or mutant PP-1*β* 3' UTR sequence (Fig. 7C). Luciferase activity analysis revealed that the luciferase activity in the PP-1*β* 3' UTR WT under miR-100-5p treatment group was markedly declined, whereas this effect was reversed in the PP-1*β* 3' UTR mutant with miR-100-5p treatment (Fig. 7D). Meanwhile, Western blot analysis revealed that the protein expression of PP-1*β* in the MI + EC-Exo group was significantly declined compared to that in the MI group (Fig. 7E, F, Additional file 1: Fig. S8). To further confirm this fine-tuned relationship between PP-1*β* and PLB, we performed siRNA-mediated gene silencing (Fig. 7G). Western blot analysis revealed that the PP-1*β* expression was significantly reduced in the siPP-1*β* group compared to the scramble group, which suggested an excellent knockdown efficiency. Importantly, p-Ser^16^-PLB presented apparent augmentation in the siPP-1*β* group compared to the scramble group, which indicated the indispensable role of PP-1*β* in regulating the PLB phosphorylation in this model (Fig. 7H, Additional file 1: Fig. S9). Overall, these results demonstrate that PP-1*β* is the specific downstream gene of miR-100-5p, and miR-100-5p enriched in the hiPSC-EC exosomes enhances the phosphorylation of PLB at Ser^16^ by targeting PP-1*β*.

### miR-100-5p plays an important role in hiPSC-EC exosome-mediated cytoprotection in improving intracellular Ca^2+^ homeostasis and cardiomyocyte survival

**Fig. 8:**
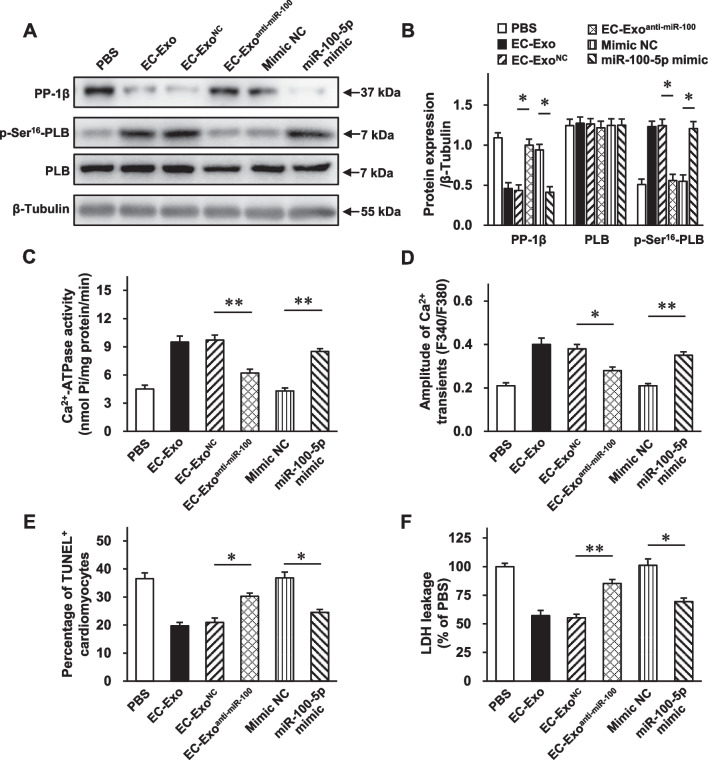
miR-100-5p loss abolishes, while miR-100-5p mimic reproduces, the part of protection of hiPSC-EC exosomes on cardiomyocytes against OGD injury. hiPSC-CMs were separately treated with PBS, mimic negative control (NC), miR-100-5p mimic, EC-Exo, EC-Exo^NC^, and EC-Exo^anti−miR−100−5p^ under OGD conditions. **A–B** Protein samples were collected from the above-mentioned groups, and Western blot was performed to assess the protein expression levels of PP-1*β*, p-Ser16-PLB, and PLB. **A** Representative immunoblots. Full-length blots are presented in Additional file 1: Fig. S10. **B** Quantitative analysis of protein expression levels. **C** SERCA activity was assessed using a Ca^2+^-pump ATPase enzyme assay kit.** D** hiPSC-CMs were incubated with a Ca^2+^ indicator (Fura-2 AM) and stimulated at 0.5 Hz; then, Ca^2+^ transients were recorded and quantified. **E** The activity of LDH released in the culture media was measured. **F** TUNEL^+^ cardiomyocytes were assessed. Quantitative data are presented as mean ± SEM. *n* = 4 independent experiments in **B** and *n* = 5 independent experiments in **C**–**F**. Significance was evaluated via one-way ANOVA followed by Tukey’s post hoc test in (**B**–**F**). *p < 0.05 and **p < 0.01

To determine the important role of miR-100-5p in hiPSC-EC exosome-mediated cardioprotection, we conducted the miR-100-5p gain–loss function experiments on cardiomyocytes under OGD injury. The Western blot analysis revealed that the dramatically declined PP-1*β* protein expression and elevated p-Ser^16^-PLB protein level in the OGD + EC-Exo group were abolished in the OGD + EC-Exo^anti−miR−100−5p^ group and reproduced in the OGD + miR-100-5p mimic group (Fig. 8A, B, Additional file 1: Fig. S10). In addition, the notably elevated SERCA activity in the OGD + EC-Exo group was mostly attenuated in the OGD + EC-Exo^anti−miR−100−5p^ group and enhanced in the OGD + miR-100-5p mimic group (Fig. 8C), which was in line with the change in Ca^2+^ transient amplitude (Fig. 8D). Furthermore, miR-100-5p loss partially abrogated, while miR-100-5p mimic partially reproduced, the effects of hiPSC-EC exosomes on reducing cardiomyocyte apoptosis (Fig. 8E) and LDH leakage (Fig. 8F). Taken together, these results demonstrate that enriched miR-100-5p contributes to hiPSC-EC exosome-exerted cytoprotective effects on increasing the PLB phosphorylation level and SERCA activity, modulating intracellular Ca^2+^ homeostasis, and promoting cardiomyocyte survival under OGD injury.

## Discussion

As a cause of congestive HF, MI is a prevalent and staggering health problem with an extensive economic burden to society. Therefore, safe and effective treatment options are urgently needed. In this study, we demonstrated the following: (i) In vitro, hiPSC-EC exosomes protected cardiomyocytes from OGD injury by impeding apoptosis, improving energy metabolism, and maintaining intracellular Ca^2+^ homeostasis, as well as enhanced the tube-forming capacity of ECs; (ii) hiPSC-EC exosome administration improved the cardiac contractile function and attenuated the harmful LV remodeling after MI without increasing the occurrence of arrhythmia; (iii) mechanistically, hiPSC-EC exosomes significantly enhanced the function and protein expression of SERCA-2a and RyR-2 to maintain cardiomyocyte Ca^2+^ homeostasis; and (iv) miR-100-5p was proven to be the most abundant microRNA and increased the phosphorylation level of PLB at Ser^16^ to improve the SERCA activity by targeting PP-1*β*, thereby contributing to the hiPSC-EC exosome-exerted cytoprotective effects on modulating intracellular Ca^2+^ homeostasis. These findings extend the previous knowledge and provide insights into the mechanisms underlying the cardioprotection conferred by hiPSC-EC exosomes, supporting the fact that hiPSC-EC exosomes may be a salutary acellular therapeutic option for MI.

The exosomes secreted by a mix of hiPSC-derived cardiac cells, containing cardiomyocytes, ECs, and smooth muscle cells, can reproduce the effects of transplanted cells and improve myocardial recovery after MI [20]. However, due to the composition complexity of hiPSC-derived cardiac cell exosomes, additional investigations are needed to elucidate which exosomes mainly contribute to the benefits against MI and the underlying mechanisms. In the present study, we first demonstrated that the administration of exosomes only from hiPSC-ECs conferred convincing cardioprotective effects characterized by amelioration of the worsened heart function, attenuation of scar formation, reduction of cardiomyocyte apoptosis, and suppression of cardiac hypertrophy. Moreover, we evaluated the safety of hiPSC-EC exosome transplantation using an isoproterenol-induced arrhythmia model and found that the delivery of hiPSC-EC exosomes did not increase the risk of arrhythmogenicity in MI mice. Mechanistically, our results demonstrated that hiPSC-EC exosomes could be directly taken up by cardiomyocytes in vitro and protect them against OGD injury via promoting cell survival, enhancing the energy metabolism, and increasing the Ca^2+^ transients, which indicated that cardiomyocytes are one of the targets of hiPSC-EC exosomes. Furthermore, our results illustrated that the secreted exosomes might be one of the fundamental mechanisms involved in the hiPSC-EC treatment in ischemic heart diseases. In addition, as fibroblasts and ECs are also among the most abundant cell types present in the mammalian heart, except cardiomyocytes [39], several experiments were performed to investigate the other potential targets of hiPSC-EC exosomes. Here, we proved that hiPSC-EC exosomes have no impact on fibroblast migration and proliferation but could regulate EC tube-forming activity, which is in line with the previous finding that hiPSC-EC exosomes promote neovascularization in a mouse model bearing ischemic limbs [40]. However, considering the length limit, detailed beneficial effects and underlying mechanisms of hiPSC-EC exosome on ECs were not further explored in this study.

It has been extensively established that disrupted Ca^2+^ homeostasis is a critical pathogenic factor in cardiac diseases, including MI-induced HF and atrial fibrillation (AF) [12, 13, 41]. Therefore, it is necessary to maintain intracellular Ca^2+^ homeostasis to alleviate the ischemic myocardial injury. Interestingly, in this study, we found the therapeutic effect of hiPSC-EC exosomes via modulation of the cardiomyocyte Ca^2+^ homeostasis. It is intriguing to find that hiPSC-EC exosome treatment can significantly enhance the Ca^2+^ transients in hiPSC-CMs under ODG injury and isolated adult mouse cardiomyocytes under MI injury. Furthermore, we found that these beneficial effects were mainly attributed to improving SERCA-2a and RyR-2 but not NCX-1 protein expression and function. Regrettably, the expression and function of L-type calcium channels (LTCC), which enable a small quantity of extracellular Ca^2+^ to enter the cytosol to trigger Ca^2+^ release from SR by RyR-2, and plasma membrane Ca^2+^ ATPase (PMCA) were not evaluated in the present study. Further studies are needed to investigate whether hiPSC-EC exosomes affect these two types of Ca^2+^-handling proteins. In addition, the positive force–frequency relationship (FFR) and calcium–frequency relationship (CaFR) are considered as hallmarks of the mature performance of cardiac tissue [6, 42, 43] because the reduction or reversal of the positive FFR and CaFR, which contributes to the contraction abnormality, is usually observed in immature and falling hearts [44]. In line with this, we observed that the amplitude of cell shortening and Ca^2+^ transients, as well as + dL/dtmax/cell resting length and + d[Ca^2+^]/dtmax/resting calcium, was significantly reversed by hiPSC-EC exosome administration in a stimulation frequency-dependent manner, which indicated that hiPSC-EC exosomes could rectify the impaired FFR and CaFR of cardiomyocytes in infarcted hearts.

Increasing intracellular Ca^2+^ levels may have implications for energy metabolism and cell apoptosis because the higher Ca^2+^ level will be taken up by mitochondria and damage its function [33]. The disruption of energy supply and utilization balances in mitochondria could result in stress and apoptotic pathway activation [33]. Our results revealed the crosstalk between Ca^2+^ homeostasis, energy metabolism, and cell apoptosis mediated by hiPSC-EC exosomes, which could ameliorate the Ca^2+^ cycling abnormalities to increase energy metabolism and diminish cardiomyocyte apoptosis. This is also supported by the evidence that gene transfer therapy to rescue SERCA-2a from failing hearts could restore its energy metabolism to a more efficient state [45]. Meanwhile, the higher intracellular Ca^2+^ level could excessively activate calpain, a well-conserved cysteine protease [46]. The hyperactivation of calpain could further degrade calcium-handling and contractility-related myofilament proteins to impair cardiomyocyte contraction and activate multiple cell apoptosis pathways [47], which is concordant with our result that hiPSC-EC exosomes could improve the recovery of SERCA-2a and RyR-2 protein expression after MI. These findings indicate that Ca^2+^ homeostasis modulation might be a molecular mechanism of hiPSC-EC exosome to ameliorate cellular energy metabolism, diminish cardiomyocyte apoptosis, and enhance cardiomyocyte contraction.

PP-1, the main abundant serine/threonine phosphatase, plays a pivotal role in regulating protein dephosphorylation and has multiple Ca^2+^-cycling protein targets, such as PLB [38]. A previous study has reported that PP-1*β* is the major catalytic subunit of PP-1 in the heart that could elevate PLB phosphorylation at Ser^16^ to enhance Ca^2+^ transients and cell shortening [38, 48]. Interestingly, in this study, we linked the inhibition of PP-1*β* to hiPSC-EC exosome-mediated Ca^2+^ modulation and cardioprotective effects. It is intriguing to observe that hiPSC-EC exosome administration inhibited PP-1*β* expression in the infarcted myocardium. This inhibition further increased the phosphorylation of PLB, which could remove its suppression on SERCA to maintain intracellular Ca^2+^ homeostasis. It has been shown that exosomes could be considered therapeutic cargoes to communicate between target cells and parental cells because exosomes are regarded as the harbor of nonsense oligonucleotides [14–16]. Therefore, another interesting observation is that miR-100-5p, the most abundant microRNA in hiPSC-EC exosomes, may serve as a novel PP-1*β* inhibitor meditating the PP-1–PLB cascade. Furthermore, the gain–loss function analysis demonstrated that miR-100-5p could contribute to hiPSC-EC exosome–provided cytoprotective effects by maintaining intracellular Ca^2+^ homeostasis and abrogating cardiomyocyte apoptosis.

Undoubtedly, there are some limitations of the present study. The present study demonstrated that hiPSC-EC exosome administration eliminated Ca^2+^ homeostasis disorders by rescuing the function and protein expression of SERCA-2a and RyR-2. However, our results only verified that miR-100-5p enriched in hiPSC-EC exosomes could reverse SERCA-2a function through the PP-1*β*–PLB axis signaling to partially favor cardiomyocyte protection. There is a need to elucidate the mechanism by which hiPSC-EC exosomes regulate RyR-2 function, as well as SERCA-2a and RyR-2 protein expression, in future.

## Conclusion

Administration of hiPSC-EC exosomes can alleviate the ischemic myocardial injury after MI, thereby restoring heart function and suppressing harmful LV remodeling by modulating resident cardiomyocyte Ca^2+^ homeostasis. Highly enriched miR-100-5p contributes to hiPSC-EC exosome-mediated beneficial effects via the PP-1*β*-PLB axis signaling pathway.

### Supplementary Information

**Additional file 1.** Supplementary figures.

## Data Availability

Data and materials used and analyzed during the current study are available from the corresponding author upon reasonable request. MicroRNA sequencing data have been deposited into the Genome Sequence Archive in the National Genomics Data Center (Nucleic Acids Res 2022) and are publicly accessible at https://ngdc.cncb.ac.cn/bioproject (project number: PRJCA013910).

## Abbreviations

hiPSC-ECs: Human induced pluripotent stem cell-derived endothelial cells
MI: Myocardial infarction
SERCA-2a: Sarcoplasmic reticulum Ca^2+^ ATPase 2a
PLB: Phospholamban
PP-1β: Protein phosphatase 1β
OGD: Oxygen–glucose deprivation
ECM: Extracellular matrix
SR: Sarcoplasmic reticulum
NCX-1: Na^+^/Ca^2+^ exchange 1
VWF: Von Willebrand factor
Ac-LDL: Dil-conjugated acetylated low-density lipoprotein
Alix: ALG-2-interacting protein X
TSG101: Tumor susceptibility gene 101 protein
hiPSC-CMs: hiPSC-derived cardiomyocytes
LDH: Lactate dehydrogenase
PBS: Phosphate-buffered saline
LAD: Left anterior descending artery
LV: Left ventricle
LVEF: LV ejection fraction
LVFS: LV fractional shortening
LVDP: LV developed pressure
LVEDP: LV end-diastolic pressure
+ dp/dt_max_: LV maximum ascending rate of pressure
−dp/dt_max_: LV maximum declining rate of pressure
WGA: Wheat germ agglutinin
TUNEL: Terminal deoxynucleotidyl transferase dUTP nick end labeling
cTnI: Cardiac troponin I
DAPI: 4′,6-Diamidino-2-phenylindole
NT: Normal Tyrode’s solution
V_max_: Maximum upstroke velocity
ATP: Adenosine triphosphate
SDS–PAGE: Sodium dodecyl sulfate–polyacrylamide gel electrophoresis
GAPDH: Glyceraldehyde-3-phosphate dehydrogenase
RT-qPCR: Real-time quantitative polymerase chain reaction
ANOVA: Analysis of variance
SPSS: Statistical Product and Service Solutions
FACS: Fluorescence-activated cell sorting
VEGF: Vascular endothelial growth factor
EC-Exo: Endothelial cell-secreted exosomes
WT: Wild type
HF: Heart failure
AF: Atrial fibrillation
LTCC: L-type calcium channel
PMCA: Plasma membrane Ca^2+^ ATPase
FFR: Positive force–frequency relationship
CaFR: Calcium–frequency relationship
BZ: Border zone
