# Supplementary material for: Exosomes secreted by endothelial cells derived from human induced pluripotent stem cells improve recovery from myocardial infarction in mice
Source: Stem Cell Res Ther. 2023 Sep 29;14:278. doi: 10.1186/s13287-023-03462-w (PMC10542240; doi:10.1186/s13287-023-03462-w)
Supplement: Supplementary file 1 — Additional file 1. Supplementary figures. [file 13287_2023_3462_MOESM1_ESM.pdf]

Figure S1

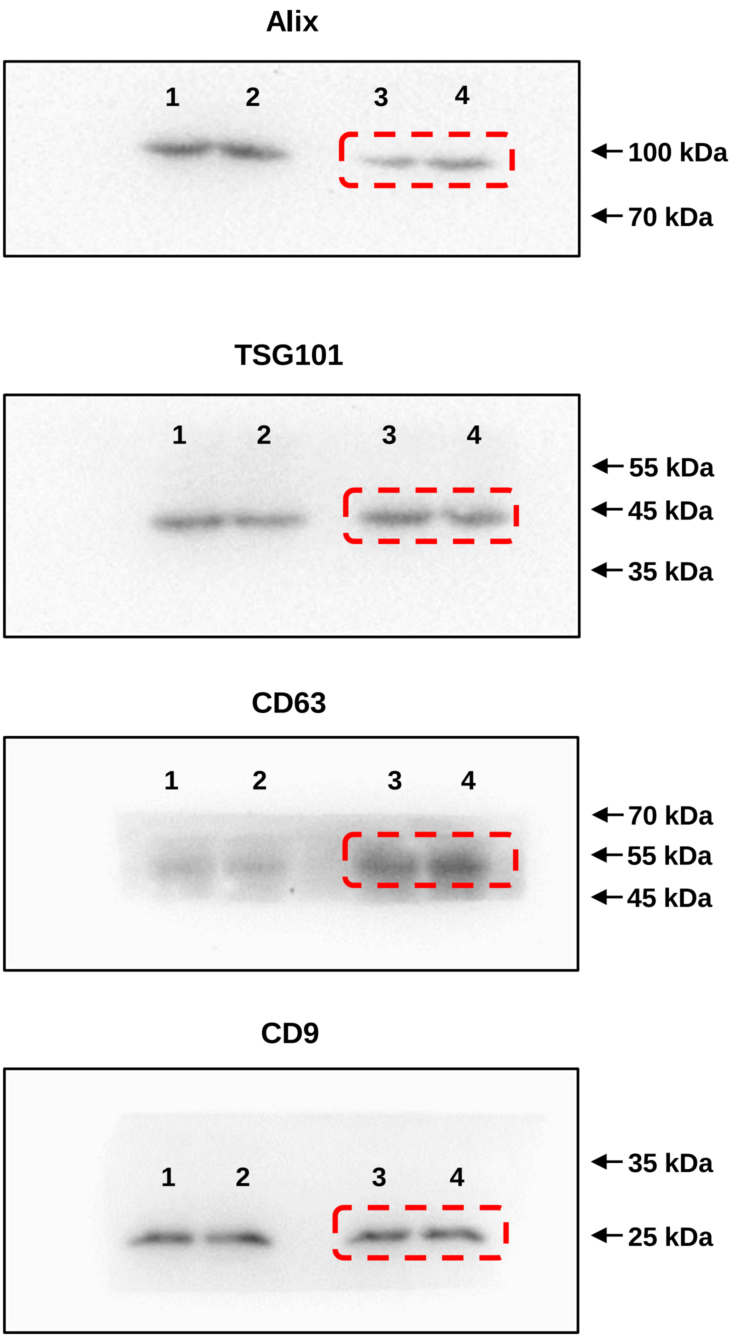

1. EC-Exo 1
2. EC-Exo 2
3. EC-Exo 1
4. EC-Exo 2

Figure S1. The unedited images of Western blot in Fig. 1I. The red boxes showed the cropped blots of the identified proteins used in Fig. 1I.

Figure S2

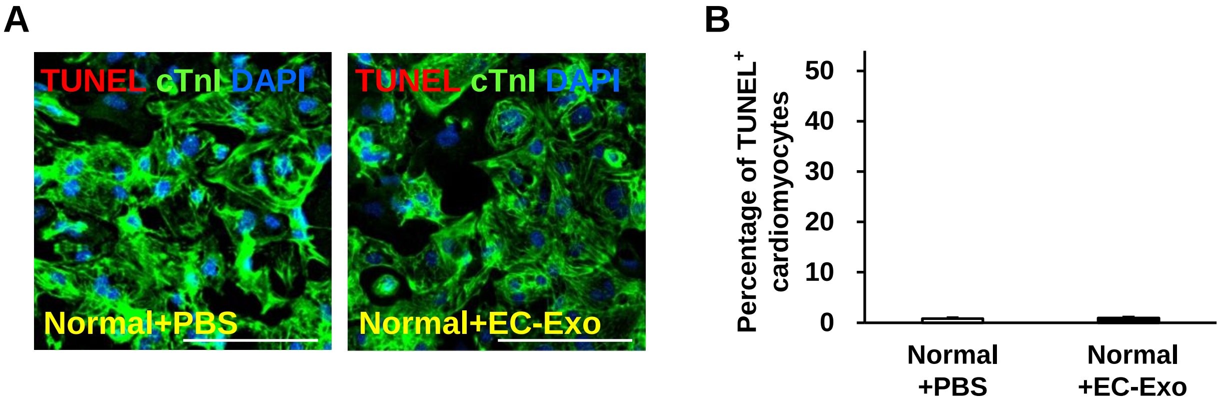

Figure S2. hiPSC-EC exosomes do not affect hiPSC-CM apoptosis under normal conditions. hiPSC-CMs were cultured under normal conditions with phosphate-buffered saline (PBS) or hiPSC-EC exosome (EC-Exo) for 48 h. (**A**) hiPSC-CMs were fixed, immunofluorescently stained for cardiac troponin I (cTnI) expression, and stained with terminal deoxynucleotidyl transferase dUTP nick end labeling (TUNEL). Then, the nuclei were counterstained with DAPI (bar = 100 μm). (**B**) Quantification of TUNEL^{+} cardiomyocytes. Quantitative data are presented as mean ± SEM, n = 4–5 independent experiments. Significance was evaluated via Student's *t*-test in (B). *$p < 0.05$.

Figure S3

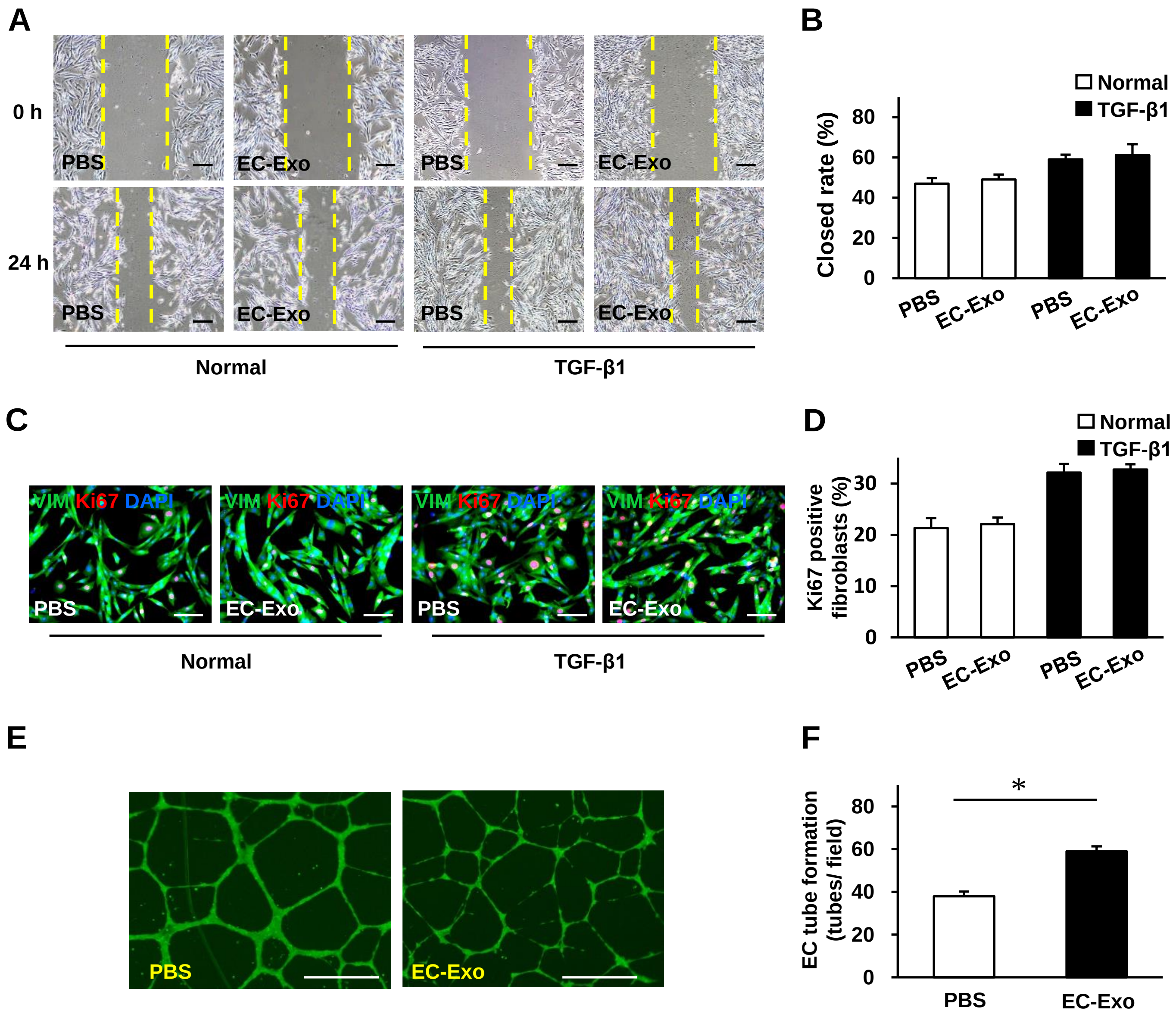

Figure S3. hiPSC-EC exosomes enhance EC tube-forming activity without affecting fibroblast migration and proliferation. (A–B) Normal or TGF-β1 (20 ng/mL)-activated fibroblasts were treated with PBS or EC-Exo in the 12-well plates for 24 h to evaluate the migration capacity (bar = 100 μm). **(A)** Representative images. **(B)** Quantification of closed rate in the wound area. **(C–D)** Normal or TGF-β1-activated fibroblasts treated with PBS or EC-Exo were immunofluorescently stained for the expressions of vimentin (VIM) and Ki67, and nuclei were counterstained with DAPI. **(C)** Representative images (bar = 100 μm). **(D)** Quantified $Ki67^{+}$ fibroblasts. **(E–F)** Endothelial cells were treated with PBS or EC-Exo for 24 h; then, the cells were labeled with calcein, and tube formation was evaluated under a light microscope. **(E)** Representative images (bar = 100 μm). **(F)** Tube formation was quantified by determining the number of tubes per field. Quantitative data are presented as mean ± SEM, n = 4–5 independent experiments. Significance was evaluated via one-way ANOVA followed by Tukey's post hoc test in (B and D) and Student's *t*-test in (F). $*p < 0.05$.

Figure S4

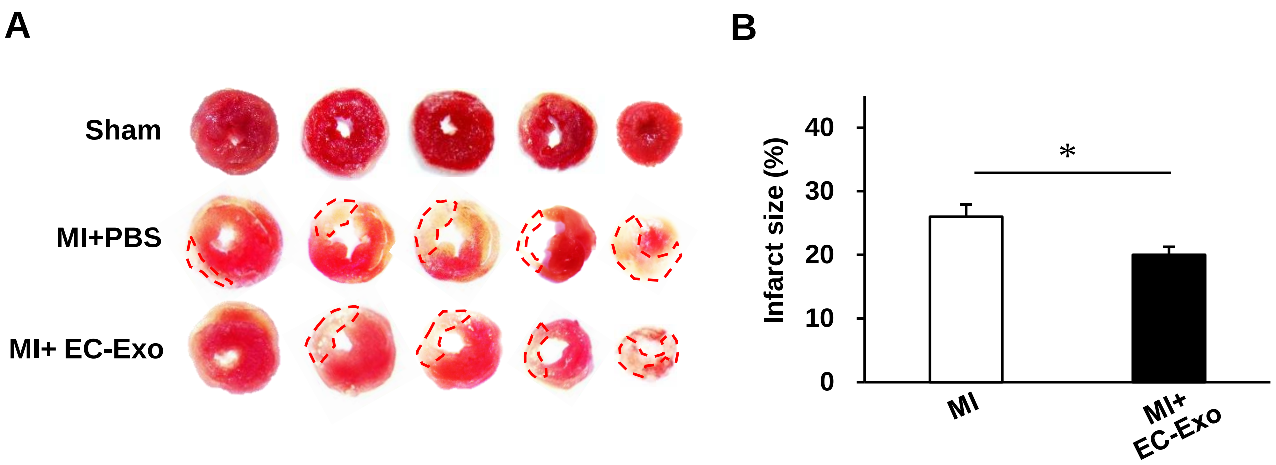

Figure S4. hiPSC-EC exosomes reduce the infarct size of mouse hearts on day 3 after MI. The mice were divided into the sham, MI, and MI+EC-Exo groups. The hearts were harvested and cut into 4-mm slices from the ligated position to the apex to evaluate the infarct size by 2,3,5-triphenyl tetrazolium chloride (TTC) staining on day 3 after MI. (**A**) Representative images of consecutive slides. (**B**) The infarct size (%) was calculated by infarct areas/total left ventricular areas. Quantitative data are presented as mean ± SEM, n = 5 per experimental group. Significance was evaluated via Student's *t*-test in (B). *$p < 0.05$.

Figure S5

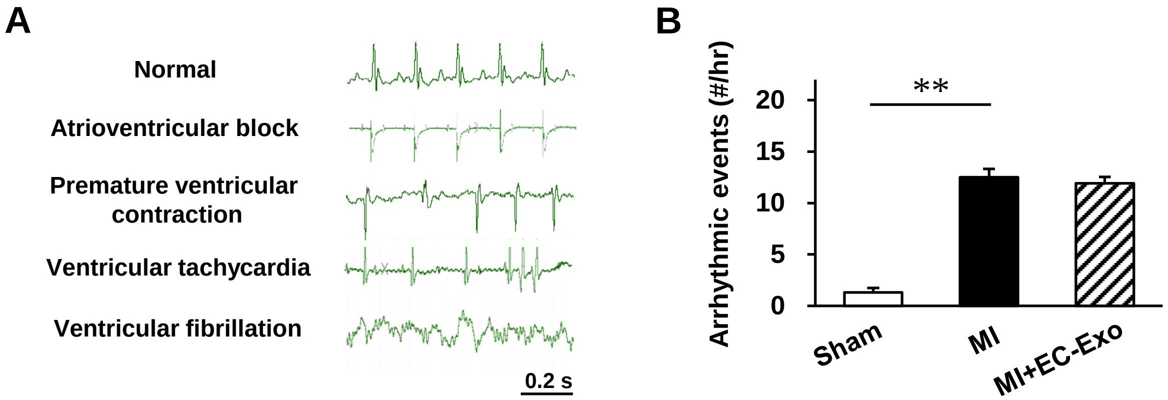

Figure S5. hiPSC-EC exosomes do not affect the incidence of arrhythmia in MI mice. Mice were intraperitoneally injected with 0.1 mg/kg of isoproterenol on day 7 after MI, and then their electrocardiograms were continuously recorded for 3 h to evaluate the incidence of arrhythmia. **(A)** Representative electrocardiograms of normal, atrioventricular block characterized by a p-wave without a subsequent QRS complex, premature ventricular contraction characterized by a widened QRS complex without a preceding p-wave, ventricular tachycardia characterized by more than three consecutive QRS complexes without preceding p-wave, and ventricular fibrillation characterized by uncoordinated electrical activity. **(B)** Arrhythmic events per hour experienced for 3 h after isoproterenol injection. Quantitative data are presented as mean ± SEM, n = 5–6 per experimental group. Significance was evaluated via one-way ANOVA followed by Tukey's post hoc test in (B). $^{**}p < 0.01$.

Figure S6

1. Sham 1
2. MI 1
3. MI+EC-Exo 1
4. MI+EC 1
5. Sham 2
6. MI 2
7. MI+EC-Exo 2
8. MI+EC 2
9. Sham 3
10. MI 3
11. MI+EC-Exo 3
12. MI+EC 3

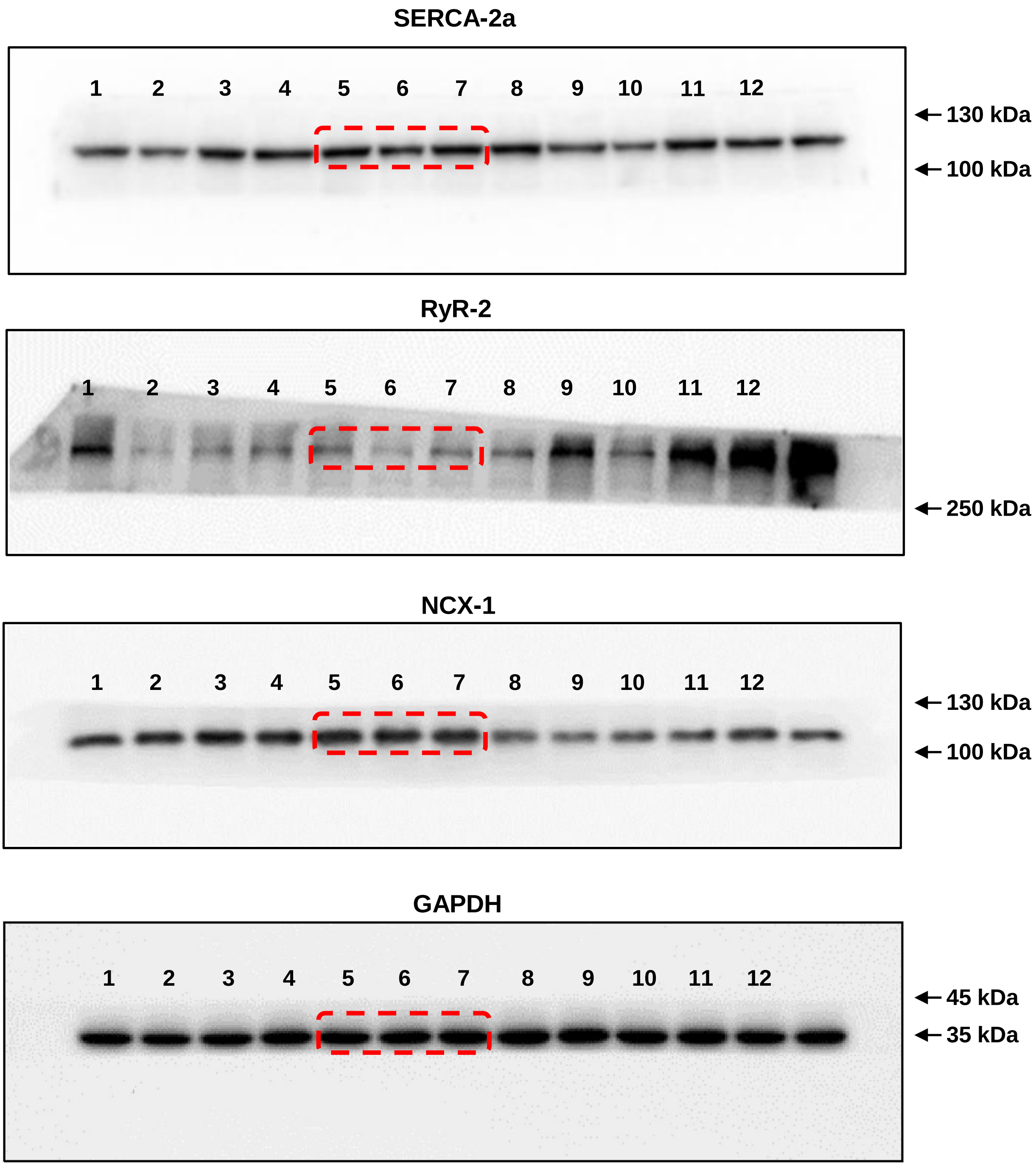

Figure S6. The unedited images of Western blot in Fig. 6A. The red boxes showed the cropped blots of the identified proteins used in Fig. 6A.

Figure S7

1. Sham 1
2. MI 1
3. MI+EC-Exo 1
4. MI+EC 1
5. Sham 2
6. MI 2
7. MI+EC-Exo 2
8. MI+EC 2
9. Sham 3
10. MI 3
11. MI+EC-Exo 3
12. MI+EC 3

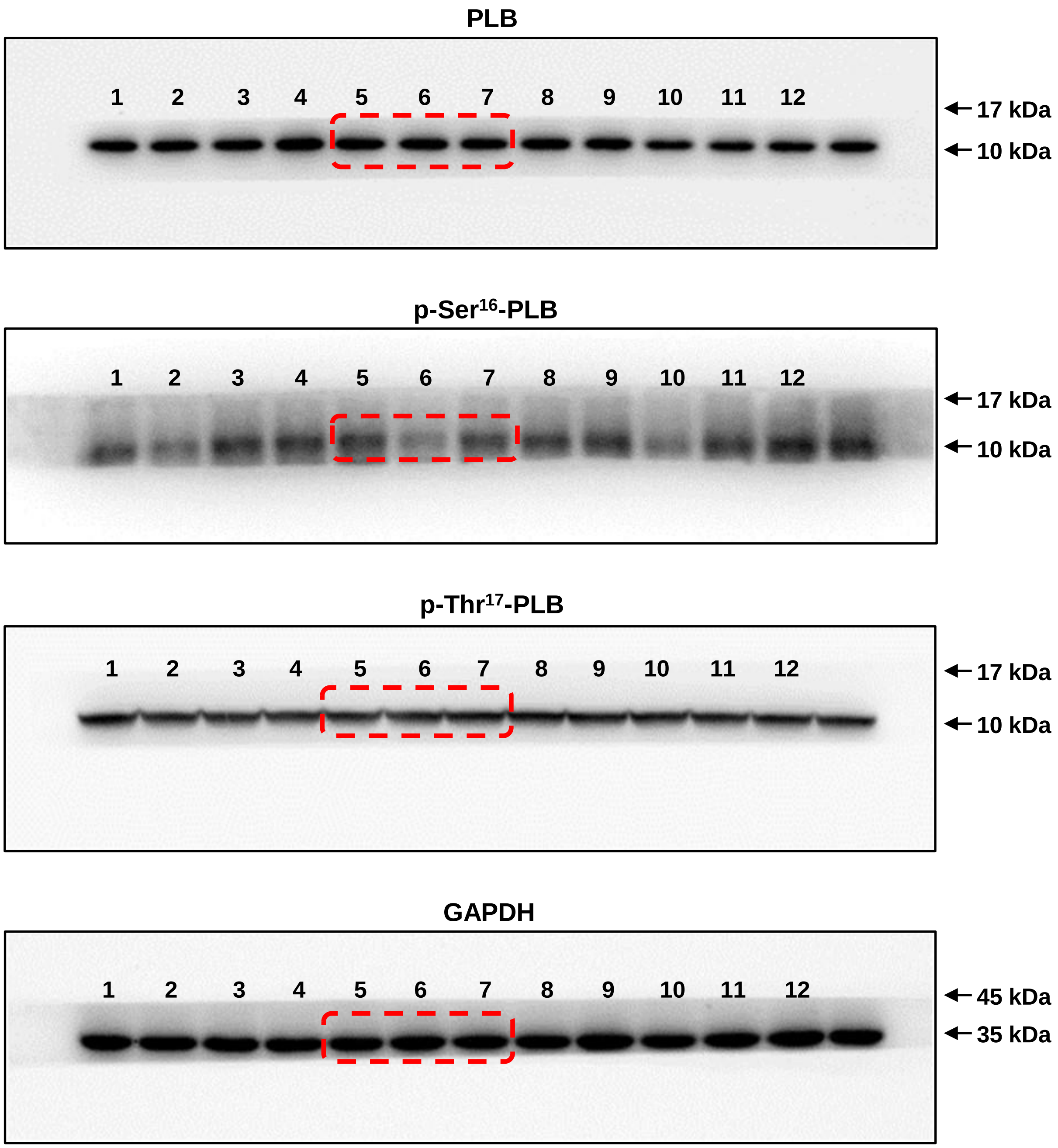

Figure S7. The unedited images of Western blot in Fig. 6C. The red boxes showed the cropped blots of the identified proteins used in Fig. 6C.

Figure S8

PP-1β

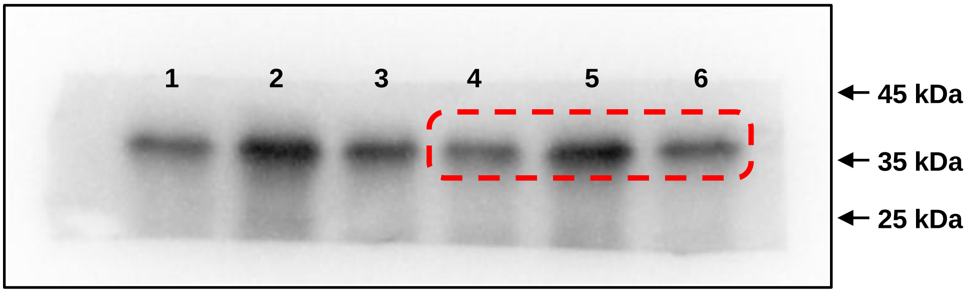

1. Sham 1
2. MI 1
3. MI+EC-Exo 1
4. Sham 2
5. MI 2
6. MI+EC-Exo 2

β-Tubulin

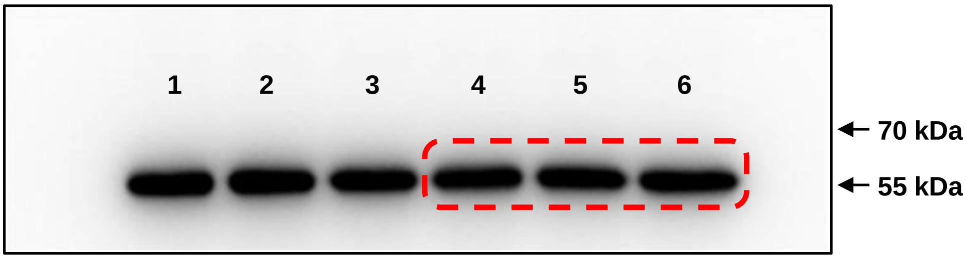

Figure S8. The unedited images of Western blot in Fig. 7E. The red boxes showed the cropped blots of the identified proteins used in Fig. 7E.

Figure S9

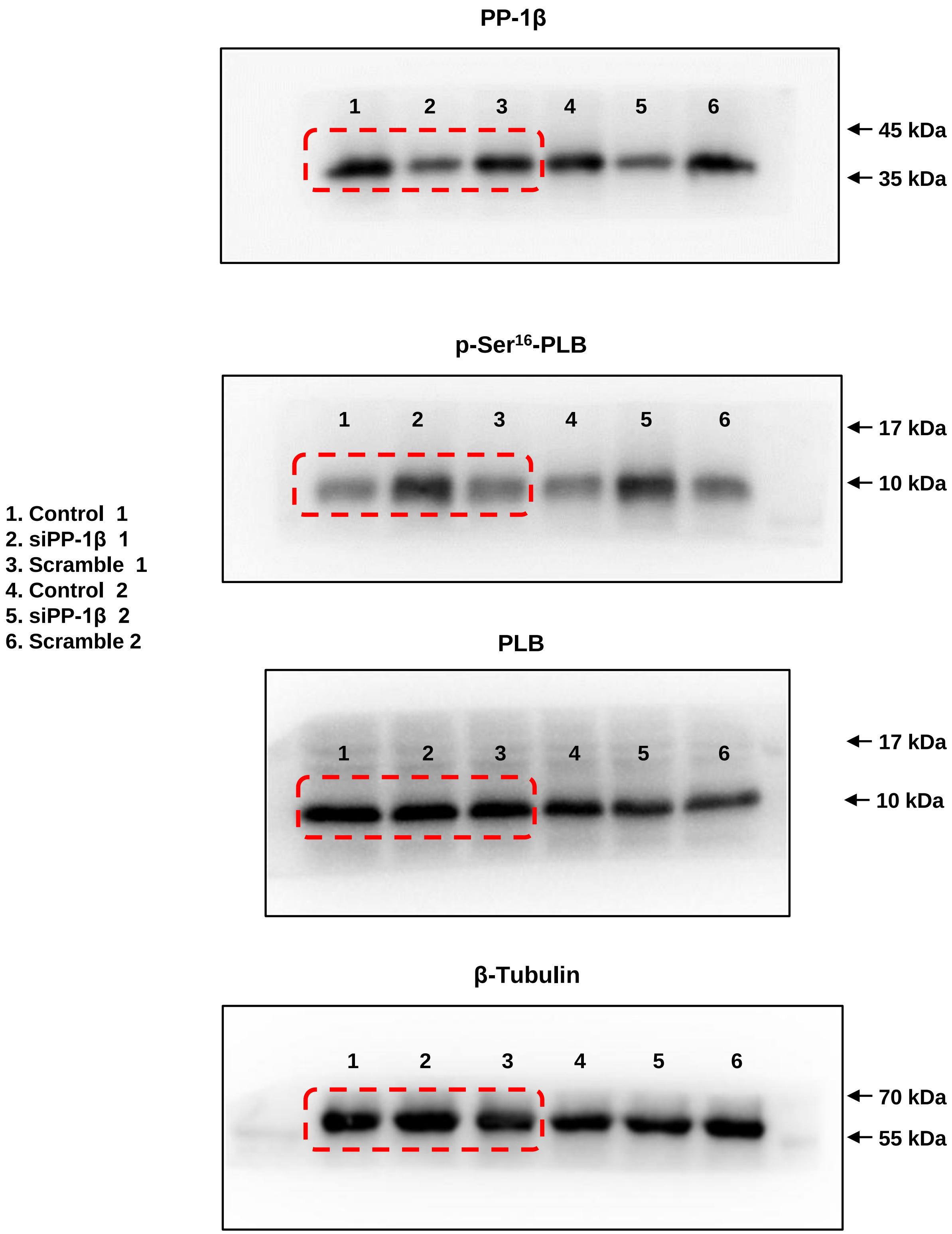

Figure S9. The unedited images of Western blot in Fig. 7G. The red boxes showed the cropped blots of the identified proteins used in Fig. 7G.

Figure S10

1. PBS 1
2. EC-Exo 1
3. EC-Exo^{NC} 1
4. EC-Exo$^{anti\text{-}miR\text{-}100}$ 1
5. Mimic NC 1
6. miR-100-5p mimic 1
7. PBS 2
8. EC-Exo 2
9. EC-Exo^{NC} 2
10. EC-Exo$^{anti\text{-}miR\text{-}100}$ 2
11. Mimic NC 2
12. miR-100-5p mimic 2

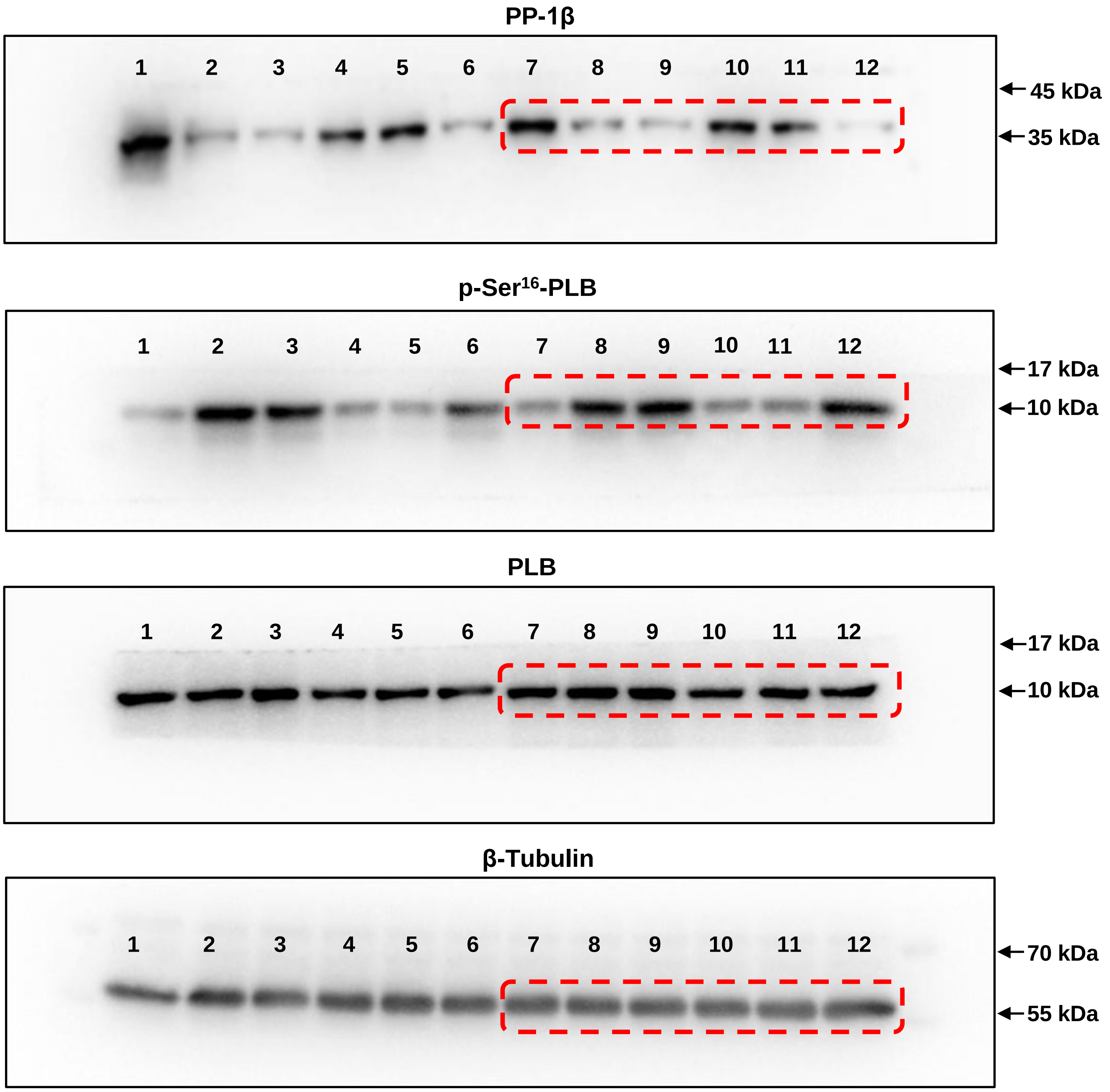

Figure S10. The unedited images of Western blot in Fig. 8A. The red boxes showed the cropped blots of the identified proteins used in Fig. 8A.
